# Spatiotemporal variability in Swedish lake ecosystems

**DOI:** 10.1371/journal.pone.0265571

**Published:** 2022-03-21

**Authors:** Tarsha Eason, Ahjond Garmestani, David G. Angeler

**Affiliations:** 1 U.S. Environmental Protection Agency, Office of Research and Development, Athens, GA, United States of America; 2 Dept. of Environmental Sciences, Emory University, Atlanta, GA United States of America; 3 U.S. Environmental Protection Agency, Office of Research and Development, Gulf Breeze, FL, United States of America; 4 School of Natural Resources, University of Nebraska-Lincoln, Lincoln, NE, United States of America; 5 Utrecht Centre for Water, Oceans and Sustainability Law, Utrecht University, Utrecht, The Netherlands; 6 Swedish University of Agricultural Sciences, Department of Aquatic Sciences and Assessment, Uppsala, Sweden; 7 The PRODEO Institute, San Francisco, CA, United States of America; 8 IMPACT, the Institute for Mental and Physical Health and Clinical Translation, Deakin University, Geelong, Victoria, Australia; Swedish University of Agricultural Science, SWEDEN

## Abstract

Studying ecosystem dynamics is critical to monitoring and managing linked systems of humans and nature. Due to the growth of tools and techniques for collecting data, information on the condition of these systems is more widely available. While there are a variety of approaches for mining and assessing data, there is a need for methods to detect latent characteristics in ecosystems linked to temporal and spatial patterns of change. Resilience-based approaches have been effective at not only identifying environmental change but also providing warning in advance of critical transitions in social-ecological systems (SES). In this study, we examine the usefulness of one such method, Fisher Information (FI) for spatiotemporal analysis. FI is used to assess patterns in data and has been established as an effective tool for capturing complex system dynamics to include regimes and regime shifts. We employed FI to assess the biophysical condition of eighty-five Swedish lakes from 1996–2018. Results showed that FI captured spatiotemporal changes in the Swedish lakes and identified distinct spatial patterns above and below the Limes Norrlandicus, a hard ecotone boundary which separates northern and southern ecoregions in Sweden. Further, it revealed that spatial variance changed approaching this boundary. Our results demonstrate the utility of this resilience-based approach for spatiotemporal and spatial regimes analyses linked to monitoring and managing critical watersheds and waterbodies impacted by accelerating environmental change.

## Introduction

Shifting ecosystem dynamics have broad implications for coupled human and natural systems. Because these systems are complex and integrated, no action exists in isolation and any disturbance may start a chain of events propagating across spatial and temporal scales [1]. Accordingly, the dynamic reality of these linked systems and potential impacts (e.g., increasing pollution levels, loss of ecosystem services, declining biodiversity), highlight the need to better understand patterns of change in social-ecological systems (SES). In the past, data availability was a significant limiting factor in these efforts, however, advancements in data collection tools and techniques have enhanced access to large-scale datasets (e.g., environmental, public health, economic). In addition, geospatial data and analytics have grown in their usefulness over the years, and data from remote sensing, drones, artificial intelligence, etc., have continued to be integrated into a variety of research areas. While access to such rich geotagged data streams provides significant opportunities for discovery and development, these data also present a challenge for researchers to capture useful information and emergent characteristics in the midst of a data deluge [2, 3]. Further, the implications of known and emerging global issues (e.g., climate change, novel viruses) necessitates the need to continue developing methods that help monitor and manage SESs in the face of accelerating environmental change.

Resilience provides a means for studying environmental change and relates to the capacity of a system to withstand change and remain within a regime with the same processes and structures [4]. While the theory has a well-documented history of development and application, the term “resilience” has many different meanings, even in ecology. Allen et al. [5] identified three main definitions of resilience for SESs to include resilience as a: rate, process or an emergent property. Resilience as a rate, also known as recovery, engineering resilience or resiliency [6], focuses on a system’s rate of return to equilibrium. Resilience as a process emphasizes building, maintaining, or enhancing system resilience of the current regime, e.g., community resilience, disaster resilience [5]. Resilience as an emergent property (e.g., social-ecological resilience) explicitly accounts for the possibility that SESs can exist in different configurations or regimes [4, 7]. Social-ecological resilience provides the foundation for a set of theories, tools and approaches for studying environmental change that examine the complex nature of ecosystems, including nonlinear change in system dynamics, tipping points and the possibility of alternate regimes in SESs [4, 8]. Here, we employ social-ecological resilience as the overarching approach to explain the dynamics of SESs, as it encompasses recovery, stability, variability, adaptation, transformation and alternate regimes, and accounts for the interconnectedness of SES on multiple spatial and temporal scales, as well as cross-scale interactions [7].

SESs have been assessed using a variety of quantitative approaches (e.g., discontinuity analyses, early warning indicators, information theory, multivariate time series modeling) which have been effective in assessing a variety of model and real, human and natural systems [3, 9–17]. These approaches examine change in system dynamics which is a critical aspect of social-ecological resilience [4, 7]. Prior research has shown that these resilience-based approaches not only detect ecosystem change but may provide warning in advance of transitions (i.e., regime shifts), possibly allowing time for management interventions [3, 11, 12, 14, 17]. While these studies primarily focused on temporal dynamics, it has been recognized that quantitative resilience studies need to be expanded to assess change in spatial dynamics at the landscape scale [18]. Research on spatial boundaries has typically resulted in the development of ecoregion maps based on ecological potential, but this method does not consider the impact of external stressors (e.g., land use, climate) on underlying system conditions [17]. These stressors may have broad implications for human and natural systems through temporal and spatial scales. Hence, further research is need on the application of techniques that evaluate ecosystem dynamics in a spatially explicit way [3, 17, 19].

Researchers have employed spatial correlates of classic early warning indicators (e.g., variance, autocorrelation, skewness) with limited success [20–26] due to a number of concerns over inconsistent results and detection accuracy [27–29], although artificial intelligence approaches may help overcome some of these problems [30]. In addition, many of these methods require temporal data to document and verify changes in results from spatial approaches [31–33]. Newer methods like recovery length and network-based indicators lack utility for assessing different system types and heterogeneous patterns, and require large, high- resolution datasets, highlighting the need for multiple methods to detect change in system dynamics [3, 34–36]. Further, many of these studies tended to focus on examining single or a few selected system variables. While this may be appropriate for models or systems that are well understood, the dynamics of real ecosystems are complex and linked, and often drivers (e.g., fast and slow variables such as flash floods or climate change) are unknown or unmeasured. Accordingly, there is a great need for methods that can track trends in multiple variables, thereby reflecting the inherent complexity in ecosystems [11, 37, 38].

A variety of multivariate methods have been used for spatial analysis including spatial variance, sequential t-test of regime shifts (STARS) and constrained hierarchical clustering to monitor spatial regimes along a rangeland transect [26, 39]. For example, Angeler et al. [40] employed multivariate spatial modelling (canonical ordination) to examine spatial pattens and functional redundancies in invertebrate community structure collected across a Swedish boreal lake landscape. Each of these approaches provides useful insights on spatial patterns, regimes and resilience that may facilitate monitoring and management. However, these methods typically require the use of multiple techniques to analyze the data (e.g., principal components analysis, redundancy analysis, variation partitioning) [26, 40].

Fisher information (FI) is a statistical measure of dynamic order in systems [41] and has been used to detect patterns in complex system behavior by examining trends in variables that characterize the state (condition) of the system [42]. The method involves simultaneously assessing the dynamics of multiple, underlying system variables by collapsing them into an index that can be used to track changes in system condition [3, 9–12, 14, 17, 43, 44]. FI has been established as a useful measure of resilience as it assesses change in complex system behavior to include the identification of ecological stability and variability, regimes and regime shifts [3, 11, 12, 14, 17, 43]. The method does not need additional analytical techniques, has no strict data requirements, does not require prior knowledge of system dynamics (3) and can handle multiple variables and a variety of data types [3, 10–12, 17]. This information theory approach has been used to examine patterns and trends in SESs [9–12, 14, 17, 44–48]; however, its application to spatial data has been limited [3, 17]. FI has previously been used to successfully detect spatial regimes in bird and zooplankton communities along transects at sub-continental and oceanic scales [17]. To transform the approach into a general method for spatial analysis, Eason et al. [3] enhanced the method to detect distinctive spatial patterns over a landscape, however the application was largely limited to simulated data at discrete time periods.

In this study, our aim is to further test the utility of FI by using it to assess spatiotemporal patterns in the biophysical condition of multiple Swedish lakes that are embedded in and form a network in a terrestrial matrix (i.e., “lake landscape”) [49]. Using readily available data supplied by the Swedish University of Agricultural Sciences, we used FI to explore spatial and temporal patterns in the physicochemical environment and phytoplankton community structure data gathered in eighty-five Swedish Lakes from 1996–2018. This study assesses whether FI can detect changes in the biophysical environment of the Swedish lakes through time and explores the implications for social-ecological resilience. Further, we examine whether there is a relationship between spatial regimes and increased variance as found in other ecosystems that are spatially explicit [17, 26] and non-explicit (summarized in Angeler et al. [50]). Specifically, we consider and analyze a northern and a southern ecoregion as two independent spatial regimes, which are separated by a “hard boundary”, the Limes Norrlandicus (information below). A recent study, based on canonical ordination, has shown that despite environmental factors steering ecological community dynamics distinctly in these two spatial regimes, the community dynamics per se were stable in a time span of approximately two decades [51]. Our study aims at complementing these previous results not only by assessing ecological patterns of system variability within both regimes, but also more specifically whether ecological dynamics change with approximation to regime boundaries, a characteristic inherent in spatial resilience and regime theory [17, 18].

## Materials and methods

### Study area and data

**Fig 1:**
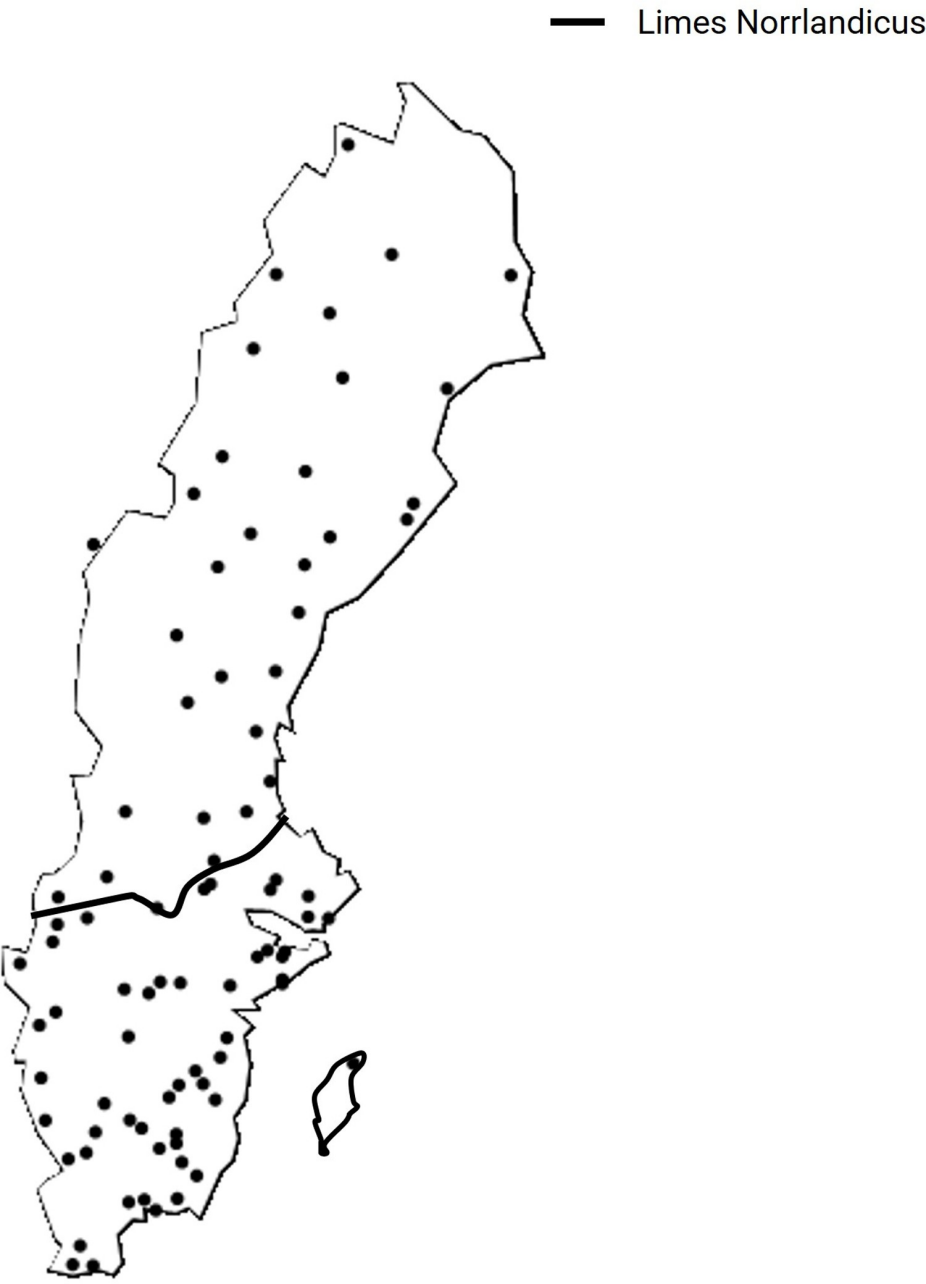
Location of Swedish lakes showing the Limes Norrlandicus (solid line).

In the late 1980s, Sweden initiated a long-term monitoring program of multiple habitats and trophic levels of lakes. Eighty-five lakes (Fig 1) with the longest continuous time series were selected from the monitoring data for this study. The lakes were situated in two ecoregions in Sweden, separated by the Limes Norrlandicus (LN), a strong biogeographical and climatic divide between northern and southern Sweden. These ecoregions differ in terms of vegetation (e.g., boreal/alpine in the north vs. hemiboreal in the south), precipitation (duration of snow cover), soil type and air temperature. Coinciding with different biotic structures, the pressures affecting the ecoregions above and below the LN are different due to higher human population density, greater agricultural land use, higher storm intensity, and historically more acidification in the southern ecoregion [51].

Despite different biophysical and anthropogenic factors characterizing the northern and southern regime at the ecoregion level (i.e., macroscale), all Swedish lakes were situated in least disturbed catchments (mesoscale). The lakes span gradients in abiotic characteristics (Secchi depth 1.1 to 10.7 m; water color <0.01 to 7.98 ±mg Pt L-1; pH 4.6 to 7.3, alkalinity <0.01 to 0.30 meq L-1; and lake size 0.11 to 5.43 km^2^). For this study, physicochemical and phytoplankton community assemblage data were compiled from data collected by the Swedish University of Agricultural Sciences between 1996–2018 (S1 and S2 Data) and used to characterize the condition of the lakes. Physicochemical variables include watercolor (absorption), water temperature (°C), pH, alkalinity (mg/L), SO_4_^2^- concentration, electrical conductivity (mSiemens/cm) and nutrients (NH_4_-N concentration (μg/L), PO_4_-P concentration (μg/L), total organic carbon (mg/L) and total phosphorus (μg/L). Data from surface water samples were selected from the periods when phytoplankton biovolume was sampled, usually in August, at a mid-lake station in each lake. Water was collected with a plexiglass sampler and kept cool during transport to the laboratory. Phytoplankton was sampled by taking a water sample from the epilimnion using a 2-m-long plexiglass tube sampler and preserving it with Lugol’s iodine-solution. Phytoplankton counts were made using an inverted light microscope and the modified Utermöhl technique commonly used in Nordic countries [52]. Specifically, taxa were identified to the finest taxonomic unit possible (usually species), and species-specific biovolume measures were calculated based on three-dimensional geometric shapes following methods employed by Blomqvist et al. [53]. All physical and chemical analyses were conducted at the Department of Aquatic Sciences and Assessment, Swedish University of Agricultural Sciences following international (ISO) or European (EN) standards, when available [54]. Additional information is available at the website of the Department of Aquatic Sciences and Assessment, Swedish University of Agricultural Sciences (http://www.slu.se/vatten-miljo) and Fölster et al. [54]. The data for this study was managed, analyzed and visualized using Microsoft Excel 365, Matlab (R2021) and JMP 14.

### Fisher information (FI)

The form of FI used for this study (Eq 1) is based on the probability of observing a particular state (condition) of the system (p(s)) [55, 56]. Given that FI is proportional to the change in probability of a particular state (dp(s)) over the change in state (ds), the higher the probability of a particular state, the more orderly (stable) the system (i.e., consistent patterns) and the higher the FI.

$$FI=\frac{ds}{p(s)}{\left[\frac{dp(s)}{ds}\right]}^{2}$$ (1)

Conversely, when a system is more disordered (highly variable) and exhibits no distinctive patterns or bias toward a particular state, FI is low and tends toward zero [57].

System states are based on the condition of the system as characterized by a set of measurable variables (s: x_1,_ x_2…._ x_n_) that may be assessed over time and/or location. Because of internal dynamics or external stressors, system condition may vary such that it experiences small perturbations or could undergo substantial change. To distinguish minor fluctuations from significant variation, a size of states (sost) is defined for each variable such that if the measured value varies within a finite range (e.g., |x_1_(t_1_)—x_1_(t_2_)| ≤ sost_x1_), the two points are indistinguishable and counted as observations of the same state. The p(s) is calculated by counting the number of points that fit within a particular state [42]. Using a series of derivation steps, Eq 1 was adapted to handle empirical data in line with this grouping (binning) approach, such that FI is calculated as shown in Eq 2:

$$FI=4\sum_{s=1}^{n}{\left[q(s)-q(s+1)\right]}^{2}$$ (2)

where q(s) ≡ √p(s) [42].

In accordance with our discussion on resilience and alternate regimes, the basis of interpreting FI is understanding that different system regimes are characterized by distinct processes and structures [4, 58]. Accordingly, tracking trends in FI affords the ability to detect change in system condition [3]. While rising FI signifies increasing dynamic order and greater stability, declining FI indicates loss of order and increasing variance which is indicative of degraded resilience in a particular regime [11, 12, 42, 44]. To compare different systems, time periods or geographical regions, basic statistical measures like the mean (μ), standard deviation (σ) and coefficient of variation (cv = $\frac{\sigma }{\mu }$) of FI are used to help classify variation in the condition to include identifying regimes and regime shifts [3, 10, 17, 59, 60]. System regimes are periods or areas in which relatively stable patterns are observed. Stable regimes exhibit more consistent patterns (greater dynamic order) and display less variability; hence, they are defined by relatively high mean FI (↑μFI), low standard deviation in FI (↓σFI) and low coefficient of variation of FI (↓cvFI). Transitional dynamics are periods where the patterns and conditions in a particular regime begin to degrade and may warn of an impending regime shift. With instability serving as the hallmark, these regime shift precursors are identified as periods with ↑σFI, ↑cvFI [3, 17]. More details on the derivation, calculation and interpretation of FI may be found in [3, 17, 42, 56, 61, 62].

### FI for spatial analysis

Using FI to assess spatial dynamics begins with compiling measurable data from each survey station. The data should include station identifiers (unique ID), location (latitude and longitude) and measured values for variables that characterize system condition (e.g., physicochemical and plankton data) over the study period: While time is a natural ordering principle for temporal studies, spatial analysis requires using a space for time substitution [17]; accordingly, Eason et al. [3] tested multiple approaches and selected Haversine distance for ordering the stations over the landscape as it most efficiently accounts for the curvature of the Earth. To order the stations, the Haversine distance is computed as [3, 63]:

$$d=2r\;arcsin\;\left(\sqrt{{sin}^{2}(\frac{\varphi_{2}-\varphi_{1}}{2})+cos\;{(\varphi }_{1})cos\;{(\varphi }_{2}){sin}^{2}\;(\frac{\lambda_{2}-\lambda_{1}}{2})}\right)$$ (3)

where, *λ* = longitude, *φ* = latitude, r = radius of the earth (r ~6.371 km) [63].

The distance is computed from a reference location (minimum latitude and longitude of the dataset). After which, the stations are sorted by distance and the data collected at the sampling stations are used to compute FI in a moving window. Each window encompasses small geographical areas over the lake landscape, such that the first window will contain the data from stations that are closest to the reference site and the second window will advance forward to the next closest station. Then, in line with the original (temporal) FI algorithm [3, 12, 17]:

- In each window, group (bin) points into states of the system using the level of uncertainty (sost) around the variables. If the measurement uncertainty for each variable is unknown, then uncertainty data may be gathered from a similar system or by using a relatively “stable” period within the dataset as a proxy. This stable period may be found by separating the data into moving windows, computing a standard deviation for each variable and using the mean standard deviation of the window with the least variation to set the sost [61].
- Count the number of points grouped into each state and divide this value by the total number of points in the window to produce *p(s)*.
- Compute *q(s) = √p(s)* and calculate FI using Eq 2.

The steps above are repeated for each moving window producing a FI value that is plotted at the corresponding sampling stations (latitude and longitude) over the geospatial area. FI code is readily accessible at https://github.com/csunlab/fisher-information [2].

### Comparative analysis

To explore possible linkages between phytoplankton community structure and changes in the physicochemical environment, we computed FI for both the phytoplankton (PHYTO FI) and physicochemical data (PC FI) over the lake landscape and compared the patterns and trends in the FI results, spatially and temporally. GIS plots and Kendall’s Tau-b correlations provided a means of visualizing the results and assessing whether the phytoplankton community dynamics are correlated with changes in the physicochemical environment. That is, we examined correlations between PHYTO FI and PC FI over the lake landscape to assess the congruence in spatiotemporal patterns between abiotic and biotic factors and explored whether distinct patterns tended to emerge above and below the LN. In addition, we assessed whether the lake ecosystem dynamics changed monotonically over time by correlating FI with the factor “year” (from 1996 to 2018) to infer whether the spatial variability of the lake landscape changed for the period of the study. Further, to help streamline the assessment, summary statistics (mean FI: μFI, standard deviation in FI: σFI and coefficient of variation in FI: cvFI) were used to examine changing condition over the landscape and through time. When identifying “stable” regimes during spatiotemporal analysis, the metrics must meet all three criteria = > ↑μFI: μFI_year_> μFI_1996-2018_, ↓σFI: σFI_year_ < mean σFI_1996-2018_ and ↓cvFI: cvFI_year_> mean cvFI_1996-2018._

## Results

The Swedish University of Agricultural Sciences dataset consists of physicochemical (PC) and phytoplankton (PHYTO) community data on 623 phytoplankton species collected at the 85 lakes between 1996–2018. These data characterize the biophysical environment of the lake landscape and were used to compute the physicochemical FI (PC FI) and phytoplankton FI (PHYTO FI). The PC and PHYTO datasets were sorted by Haversine distance in each year and stored in separate workbooks (S1 and S2 Data).

**Fig 2:**
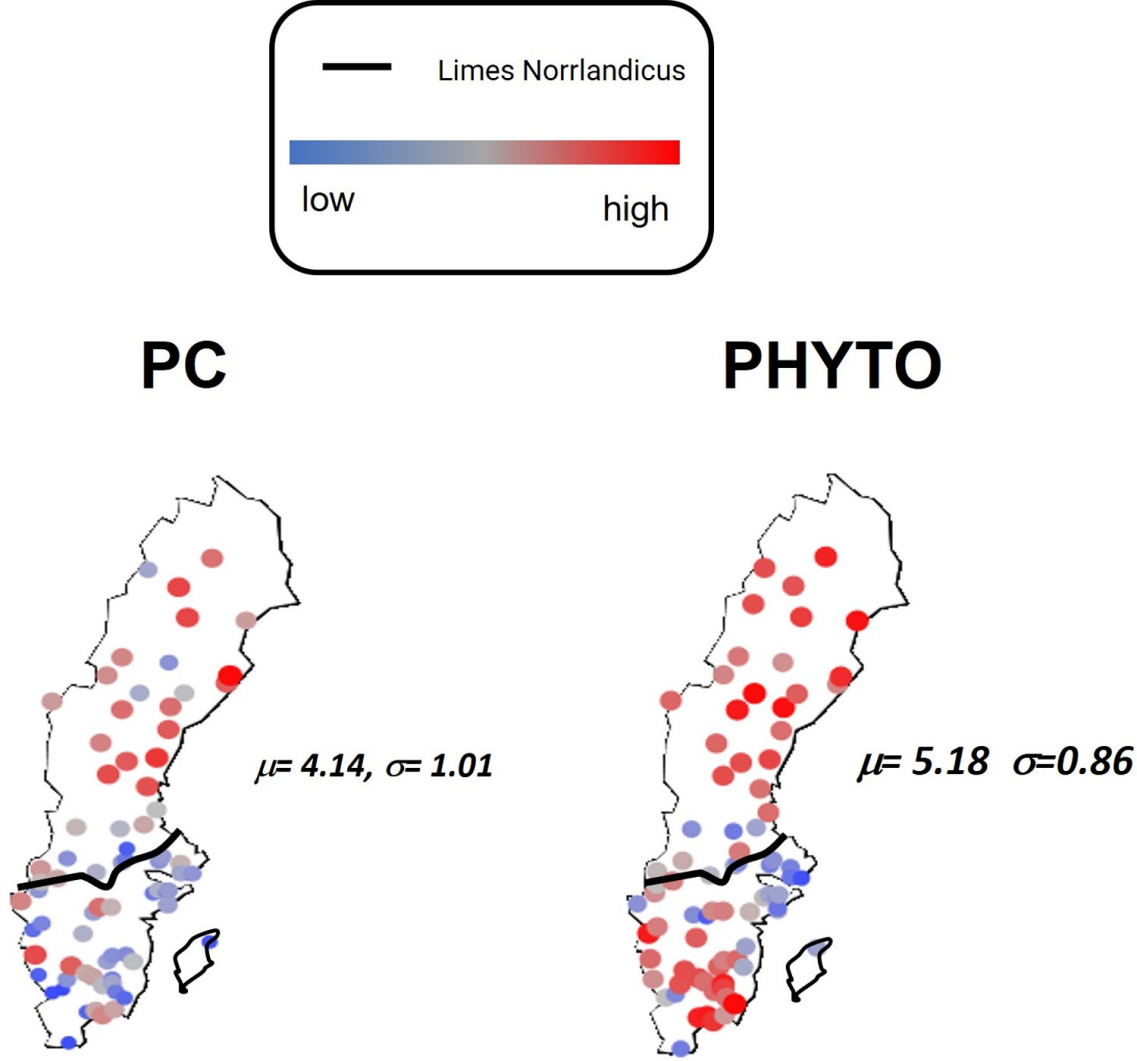
GIS plots of the 1996 FI results computed from the physicochemical (PC) and phytoplankton (PHYTO) data. A blue to red color scheme and marker size gradient are used to distinguish low to high FI values over the landscape.

Using data from the first year of the study (1996) and a window size of 10 (i.e., 10 lakes), we calculated the PC FI and PHYTO FI which were plotted at the lake locations (latitude and longitude) corresponding to each window. Fig 2 provides GIS plots of the results using color and marker size gradients to differentiate low (blue) and high (red) FI values and help visualize the changing biophysical condition over the lake landscape. While FI values tended be higher above and decline approaching the LN for both datasets, the LN served as a much clearer demarcation for lower PC FI values. In addition, we found that the PC FI and PHYTO FI values were positively correlated (τ: 0.32, p-value <0.05) with the PC FI values which displayed more variability (higher σFI (PC: 1.01 > PHYTO: 0.86) and higher cvFI (PC: 0.24 > PHYTO: 0.17)). GIS plots of the PC FI and PHYTO FI from 1996–2018 are provided in the (S1–S6 Figs). To examine how these spatial patterns emerged and changed over time, we used Kendall’s Tau correlations and summary statistics (μFI, σFI and cvFI) to streamline the comparisons and facilitate the assessment.

### Assessing spatiotemporal change

**Fig 3:**
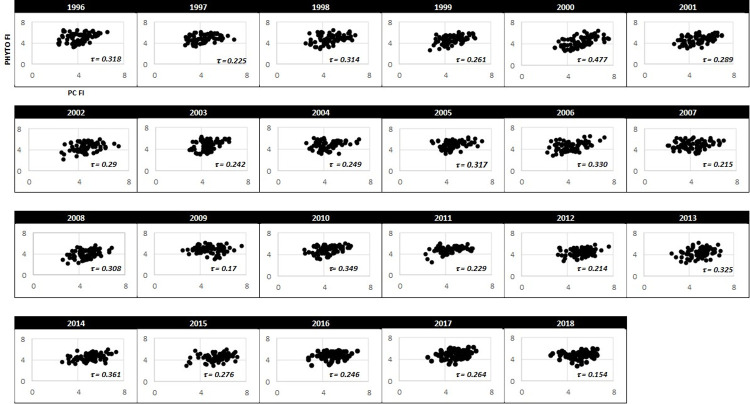
Scatterplots of PHYTO FI vs PC FI showing the Kendall’s Tau correlation value (τ, p-value<0.05) for each year of the study.

**Fig 4:**
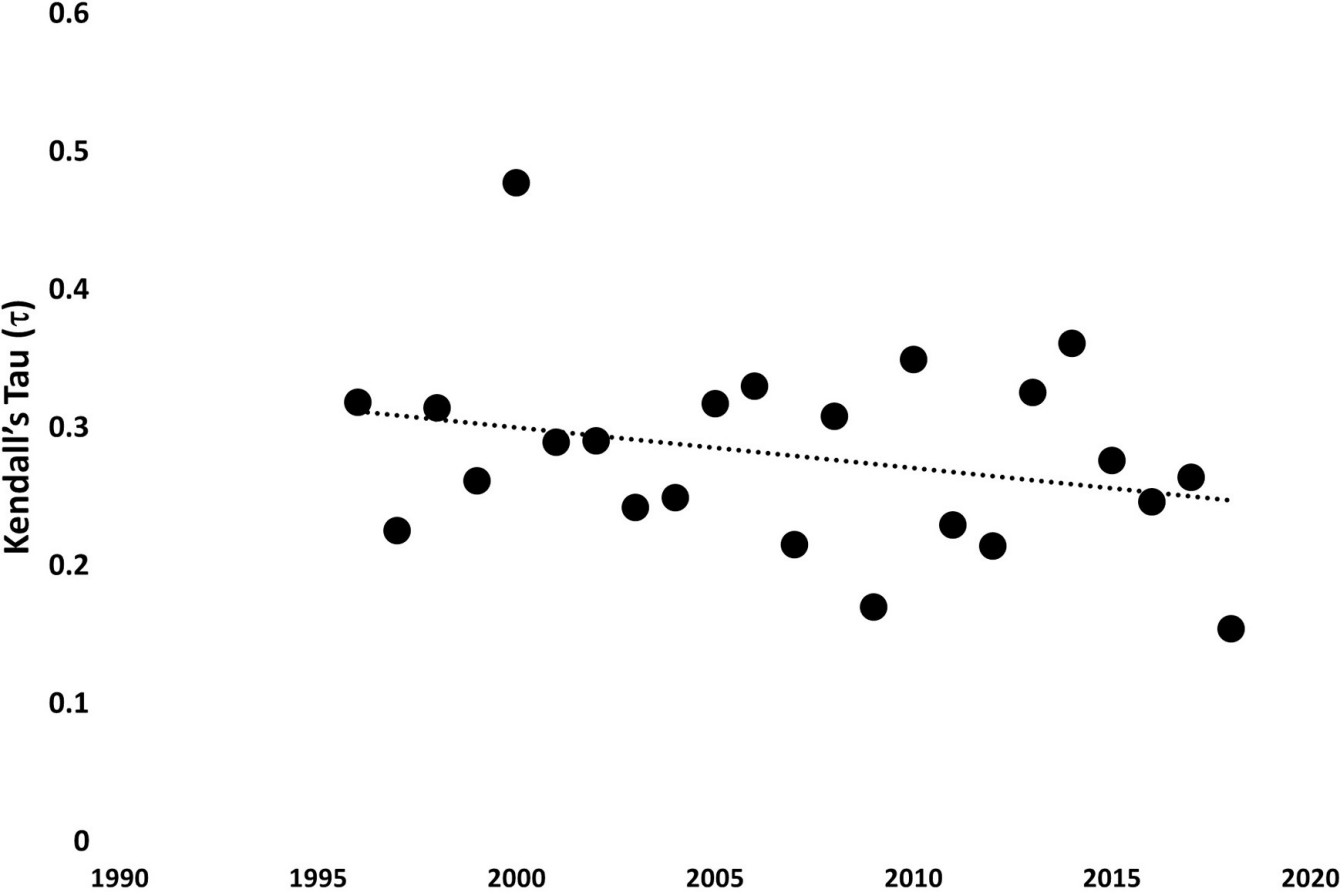
Kendall’s Tau correlation values (p-value<0.05) between PHYTO FI and PC FI indicate a decline in the connection between phytoplankton and physicochemical dynamics over time.

**Fig 5:**
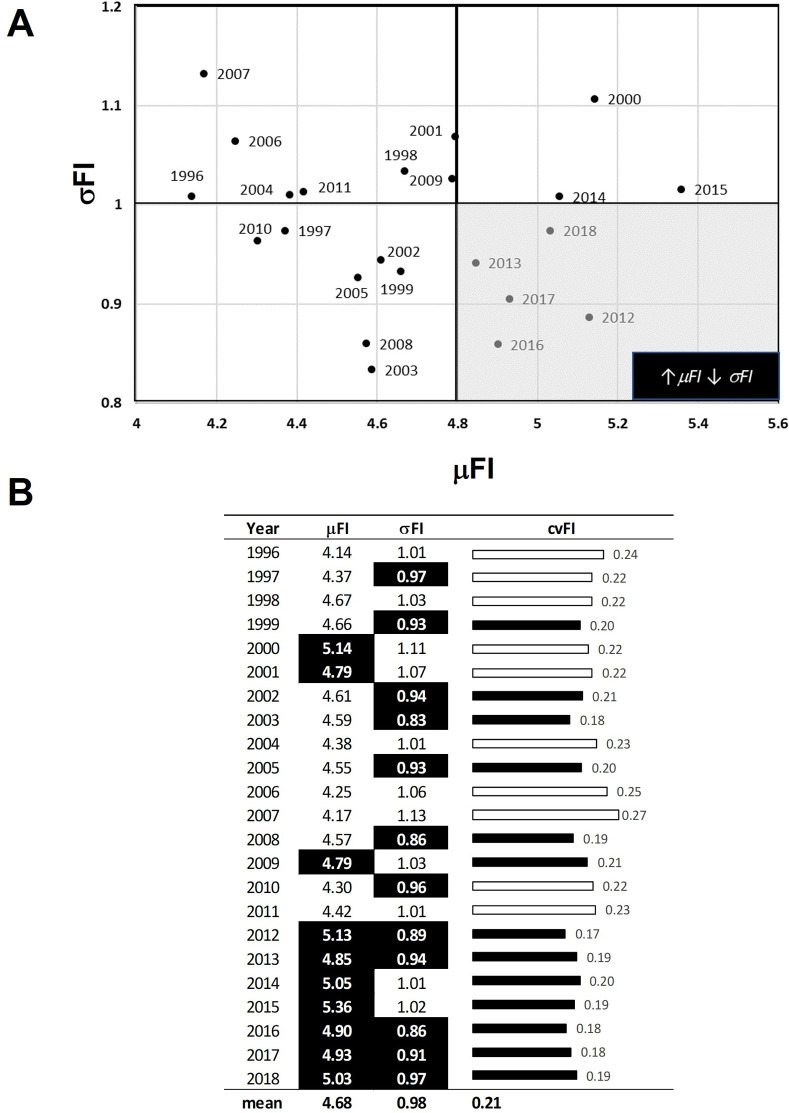
Summary statistics on the spatial variation in physicochemical (PC) dynamics. (A) The μFI and σFI plot helps identify more stable (less variable) time periods which are characterized by high μFI, low σFI and cvFI (low right corner). (B) Summary statistics for each year including the cvFI. Highlighted values are those meeting the “stable” regimes criteria for the individual measures (↑μFI, ↓σFI and ↓cvFI).

**Fig 6:**
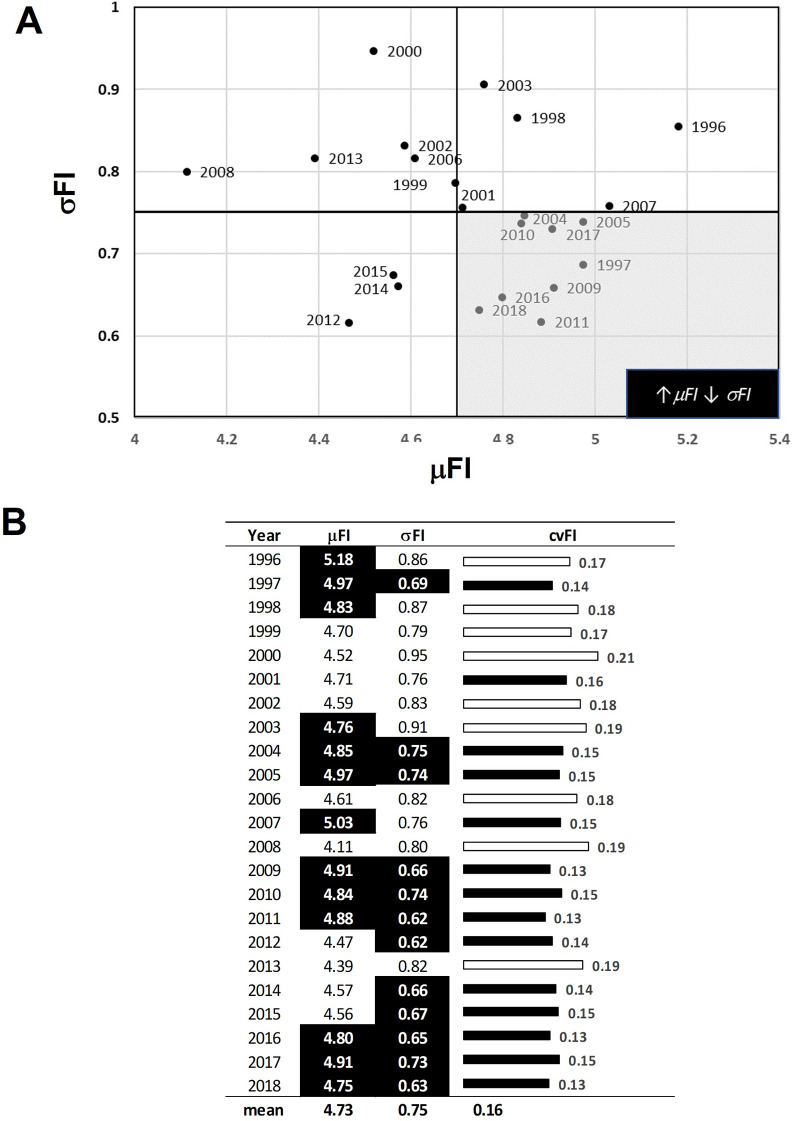
Summary statistics on spatial variation in phytoplankton community (PHYTO) dynamics. (A) The μFI and σFI plot helps identify more stable (less variable) time periods which are characterized by high μFI, low σFI and cvFI (low right corner). (B) Summary statistics for each year including the cvFI. Highlighted values are those meeting the “stable” regimes criteria for the individual measures (↑μFI, ↓σFI and ↓cvFI).

**Fig 7:**
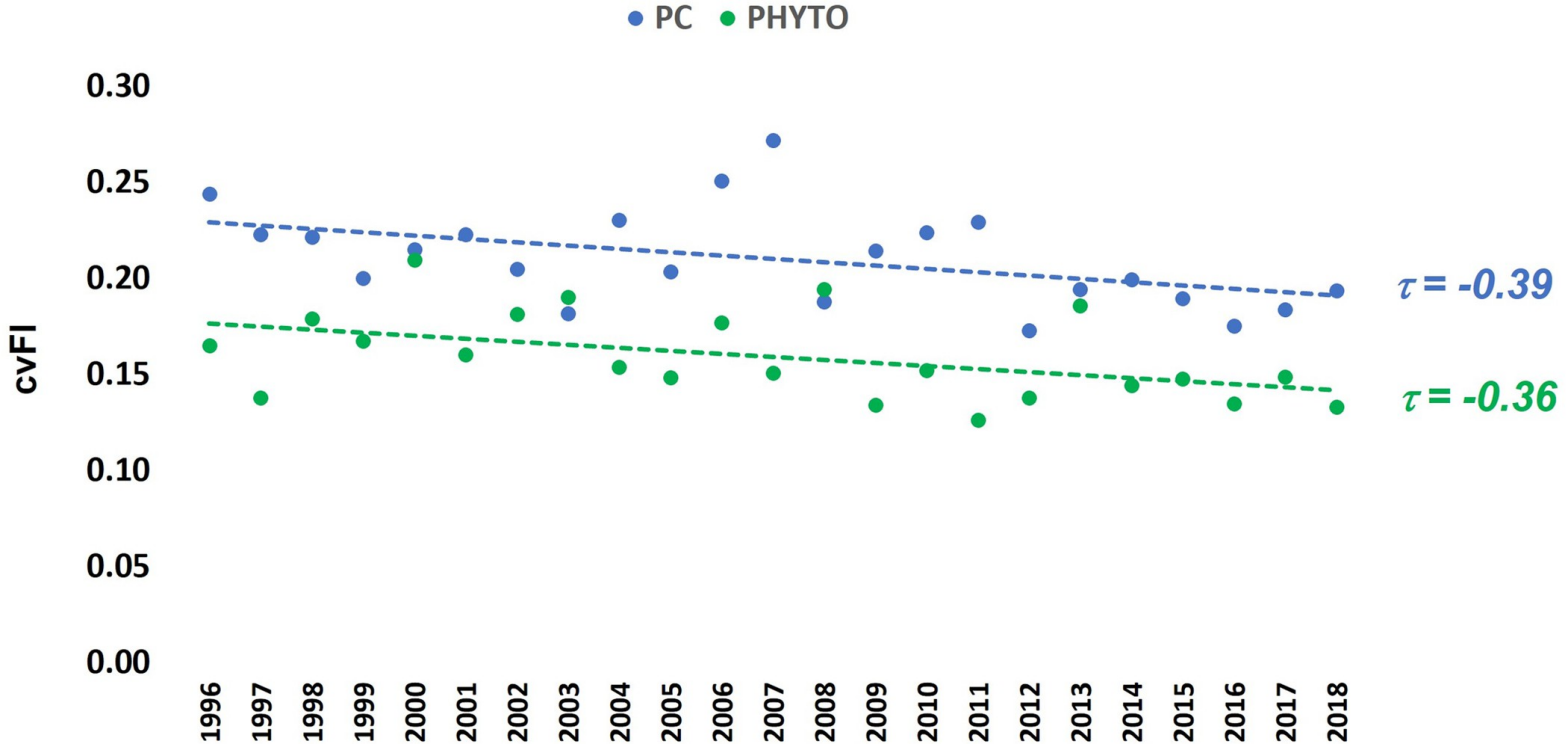
Spatiotemporal variation of the PC and PHYTO dynamics. The cvFI serves as a measure of variation around the mean; hence, lower cvFI values indicate increasing stability. The statistically significant Kendall’s tau correlation values (p-value < 0.05) show the strength of the trend.

Scatterplots in conjunction with Kendall’s Tau correlations showed that the PC FI and PHYTO FI values at least moderately corresponded over time (Fig 3). The strength of the association ranged from weak (τ_2018_ = 0.15, p-value<0.05) to strong (τ_2010_ = 0.48, p-value<0.05) and declined during the study period (Fig 4). Figs 5 and 6 provide the summary statistics which help capture changes in spatial variation for PC and PHTYO dynamics over time. The most stable years are found in the lower right-hand side of the μFI vs σFI plot (↑μFI, ↓σFI; Figs 5A and 6A) and in Figs 5B and 6B, the highlighted values are those meeting the “stable” regimes criteria for the individual measures (↑μFI, ↓σFI and ↓cvFI). Given the higher μFI and lower σFI and cvFI, there was greater stability (less variability) in PHYTO FI. In addition, there was greater congruence in the stability criteria for PHYTO FI relative to PC FI (PC: 2012, 2013, 2016, 2017 and 2018; PHYTO: 1997, 2004, 2005, 2009–2011, 2016, 2017 and 2018) with both experiencing more stable patterns near the end of the study period (2016–2018) (Figs 5B and 6B). Fig 7. provides a plot of the cvFI for PC FI and PHYTO FI over time. Since cvFI is a measure of dispersion around the mean, the cvFI alone is a useful aggregate for capturing the spatial variation over the lake landscape, such that higher cvFI values indicate greater variation. Results show statistically significant declines in cvFI for both PC (τ_PC_: -0.39, p-value < 0.05) and PHYTO FI (τ_PHYTO_ = -0.36, p-value < 0.05) and lower cvFI values for PHYTO over the entire period indicating greater stability.

**Fig 8:**
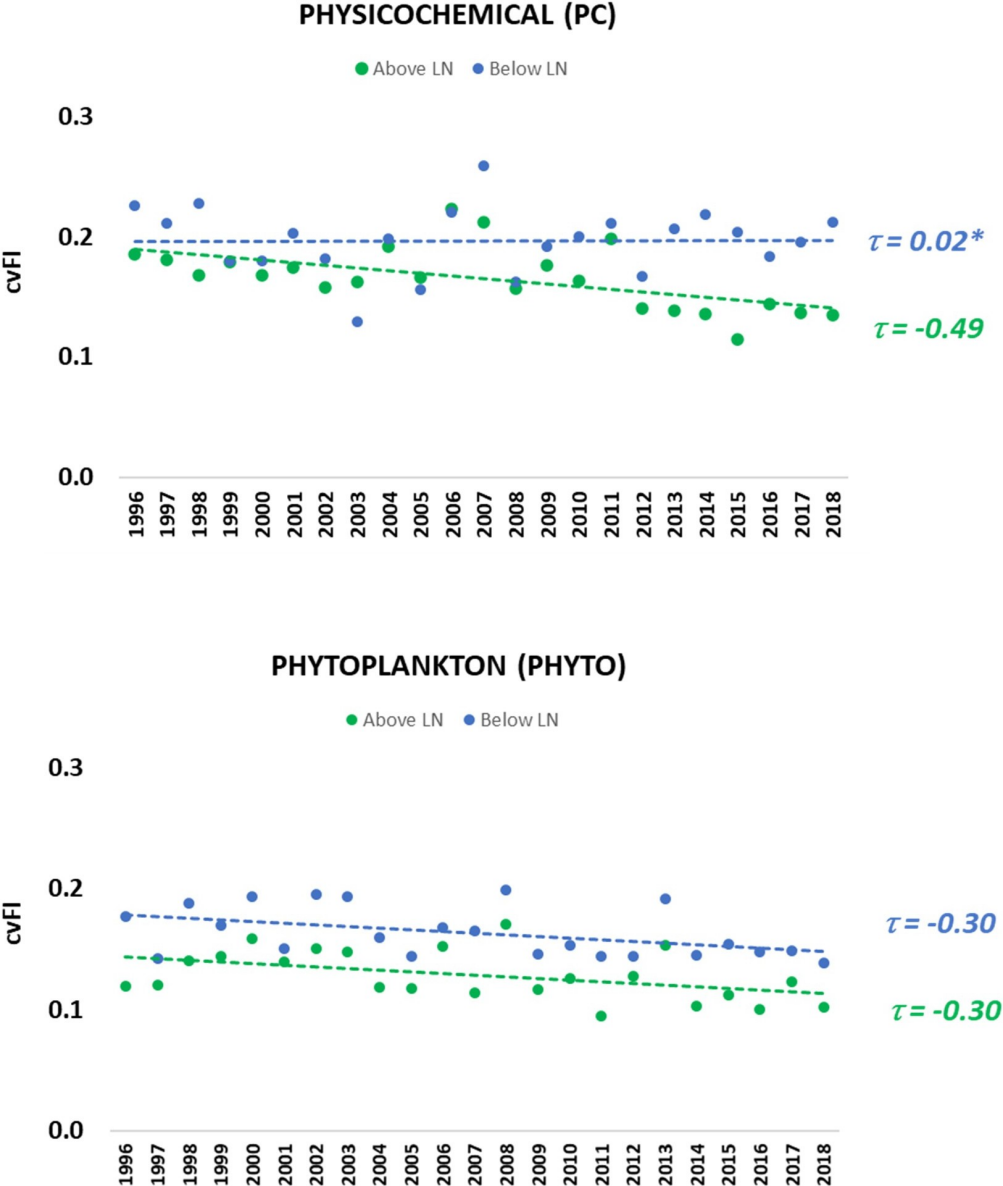
Spatiotemporal variation in biophysical condition above and below the Limes Norrlandicus (LN). The cvFI serves as a measure of variation around the mean; hence, lower cvFI values indicate increasing stability. The statistically significant Kendall’s tau correlation values (p-value < 0.05) show the strength of the trend. Note: *indicates that the correlation was not statistically significant (p-value>0.05).

To examine the difference in spatial variability between Sweden’s northern and southern ecoregions, we partitioned the data and computed the cvFI on the lakes residing above (N = 31) and below (N = 54) the LN. Results showed that the cvFI values were higher below the LN indicating greater variability (Fig 8). Further, we found evidence of declining trends in cvFI for PC and PHYTO above the LN and PHYTO below the LN (t ≤ -0.30, p-value <0.05).

## Discussion

Lake ecosystems vary temporally, spatially and across scales. Although they are typically able to maintain processes, structures and functions, accelerating environmental change can erode lake resilience, reducing their capacity to withstand disturbances, and resulting in undesirable consequences (e.g., regime shifts to eutrophic lakes). Social-ecological resilience presents a means of understanding SES dynamics and accounts for the fact that natural systems rarely change monotonically, instead they are often characterized by ecological thresholds, nonlinear change and alternate regimes [4]. Resilience-based approaches have proven to be powerful tools for examining coupled human and natural systems. One such method is FI, however, there has been limited application of this approach to assessing spatial dynamics [3, 9–12, 14, 17, 44, 47]. This work serves as the first time that FI was used to study spatiotemporal dynamics at the landscape scale. Using the approach, we were able to assess variation in the biophysical conditions of the Swedish lakes landscape, which was useful for gaining better insight into the dynamics of these lakes from the perspective of spatial resilience and spatial regimes.

To evaluate patterns of stability and variation within the Swedish lakes landscape from the viewpoint of spatial regimes [18], this study involved compiling data on the physicochemical environment and phytoplankton community structure of 85 Swedish Lakes from 1996–2018 and determining whether FI was able to detect spatiotemporal change in biophysical conditions across the Swedish lake landscape. Results showed that FI values tended to be higher with less variation at sampling stations in the northern spatial regime relative to the southern spatial regime which suggests that the condition of lakes in the northern spatial regime is more stable both spatially and temporally (Figs 2, 8, S1–S6 Figs). This finding is in agreement with a previous study based on benthic macroinvertebrates, which showed that differences in stability were influenced by different environmental variables [51]. The PC and PHYTO data displayed distinct levels of variation with more spatial stability found in phytoplankton community structure, suggesting greater spatial resilience. However, there was more congruence and less variability in biophysical condition near the end of the study period (Figs 5–7). This observed congruence between results of phytoplankton and physicochemical conditions may be useful for management. There is a rich body of ecological literature which explored the congruence of patterns between taxonomic groups as indicators of environmental change and ecological status (e.g. [64]), which suggested that weak congruence between results from different ecological communities requires multiple lines of evidence to help strengthen the inference [65–67]. Given that monitoring lakes to the extent done in the Swedish lakes monitoring program is extremely costly, our results suggest that measuring either phytoplankton or the physicochemical environment may lead to similar results about spatial and temporal variability in the lake landscape; thereby, substantially reducing monitoring costs. However, we emphasize that such substitutions may not be generalizable and need to be considered with caution and more research.

Our findings showed that while the biophysical conditions of the lakes generally increased in stability over time. Physicochemical conditions and phytoplankton community structure varied from year to year and experienced a lengthy period of instability before both appeared to find more consistent patterns in 2016 (Figs 5–7). Such a monotonic trend fits the pattern observed in previous research which found that spatial phytoplankton community turnover (i.e., the spatial differentiation of community structure) increased while nestedness (a surrogate for species extinctions) decreased linearly over time [68]. Community stability despite higher community turnover has also been observed in other studies (e.g., [69]). Other research focusing on the abiotic environment has shown linear changes in decreasing total phosphorus concentrations across lakes due to climate change and recovery from acidification [70]. Whether the increasing phytoplankton spatiotemporal resilience observed in this study is linked to changes in regional nutrient patterns or other factors remains to be explored.

Focusing in on the spatial regimes [17] aspect of our study, our analysis of the spatial dynamics above and below the Limes Norrlandicus (LN) identified patterns of decreased spatial variation (greater stability) in biophysical conditions (both PC and PHYTO) in the vicinity above the LN and below the LN in PHYTO dynamics (Fig 8). The analysis revealed a spatial transition zone (↑σFI and ↑cvFI) which coincides with the ecotone boundary that separates the southern and northern biogeographical areas of Sweden (Fig 2; S1–S6 Figs). The results of our analysis are in agreement with changes in community structure previously observed in lake invertebrate communities [71], and delineating the northern and southern biogeographical areas as spatial regimes [17], based on their biophysical and climatic status (see Methods). These results are also supported by analyses finding differences in the correlations between ecological stability patterns and environmental variables [51]. Lower variation in dynamic order as captured by cvFI seems to indicate spatial resilience as a function of transitions between spatial regimes in the Swedish lakes landscape. These changes differ between ecoregions that are separated by a clearly delineated stable boundary in the Swedish landscape [51], the Limes Norrlandicus [72]. Therefore, from a spatial regimes perspective, our results provide empirical support of non-random patterns of variability at the edge of regimes which has been discussed in ecological resilience [73]. Researchers have found that variables like animal fitness, nomadism and species invasions are variable at ecological transition zones [50]. Our study is, to the best of our knowledge, among the first assessing such non-randomness in an explicit spatial context by providing evidence for the assumption that higher variability manifests at spatial transition zones, and that spatial resilience might decrease towards spatial regime boundaries [18]. While our study assessed spatial variability in relation to a stable biogeographical frontier, a recent study has shown how spatial regime boundaries can be assessed when they change dynamically as a function of land-scape level changes in vegetation cover and animal communities [39].

Results also revealed a decline in the correlation between PC and PHTYO dynamics indicating an increased decoupling over time. Phytoplankton biodiversity and community structure is largely shaped by water chemistry (particularly nutrients) but can also be influenced by biotic interactions and spatial effects at the inter-basin scale and the resulting spatially structured abiotic lake environment (e.g. [67]). Therefore, there is high complexity mediating community dynamics that is difficult to scrutinize; accordingly, at this stage, we can only speculate about likely effects of biotic interactions influencing phytoplankton in a way to potentially explain the increasing decoupling over time. It is also speculative to relate an increasing occurrence of blooms and the spatial expansion of the raphidophycean flagellate, *Gonyostomum semen*, to heightened variability in community dynamics [74] affecting the observed correlation patterns. Our resilience-based study builds upon the evidence provided by more traditional analyses used in ecology. Specifically, the successful application of FI is supported by a high congruence between the identified FI patterns and the spatial structure revealed in traditional [71] and resilience-based analyses of littoral macroinvertebrate assemblages [40].

In line with our results, researchers have found evidence that despite the long-term changes in the abiotic and biotic environment observed, Swedish lakes have been quite stable thus far [75], meaning that there is no evidence that the entire lake landscape is at eminent risk of a broad-scale, regional regime shift. Investigations about the contribution of individual lakes to the stability of the lake landscape have started recently and are thus preliminary [51]. Additional studies and other methods can complement our work by helping to determine the specific conditions that changed in the SES, and underlying causes. As more spatial data accumulate, future work could aim to better link resilience-based approaches to real world applications of spatial resilience and spatial regimes detection by further examining index results to uncover trends in underlying ecological variables and identifying key drivers useful for monitoring and management. Such efforts are warranted given that accelerating environmental change substantially alters the configuration of and shifts between spatial regimes [76, 77].

We conclude by highlighting that the monitoring data only provided us with the opportunity to analyze data from a single month (usually August). While providing important insight into patterns of spatial variability, we acknowledge that phytoplankton community structure can undergo substantial seasonal change. Our results are therefore only relevant for the summer and should not be generalized for other seasons. Notwithstanding, our analyses support the value and power of a resilience-based approach (FI) for assessing spatiotemporal change in SES. Further development and application of such approaches would provide more focused and relevant information to support systems-based management and policy decisions and facilitate efforts to manage social-ecological resilience in the face of rapidly accelerating environmental change.

## Supporting information

S1 Fig GIS plots of the Fisher information results computed from the 1996–2003 physicochemical data. A blue to red color scheme and marker size gradient are used to distinguish low to high FI values over the landscape and the mean (μ) and standard deviation (σ) of FI are shown next to each plot.
(TIF)

S2 Fig GIS plots of the Fisher information results computed from the 2004–2011 physicochemical data. A blue to red color scheme and marker size gradient are used to distinguish low to high FI values over the landscape and the mean (μ) and standard deviation (σ) of FI are shown next to each plot.
(TIF)

S3 Fig GIS plots of the Fisher information results computed from the 2012–2018 physicochemical data. A blue to red color scheme and marker size gradient are used to distinguish low to high FI values over the landscape and the mean (μ) and standard deviation (σ) of FI are shown next to each plot.
(TIF)

S4 Fig GIS plots of the Fisher information results computed from the 1996–2003 phytoplankton data. A blue to red color scheme and marker size gradient are used to distinguish low to high FI values over the landscape and the mean (μ) and standard deviation (σ) of FI are shown next to each plot.
(TIF)

S5 Fig GIS plots of the Fisher information results computed from the 2004–2011 phytoplankton data. A blue to red color scheme and marker size gradient are used to distinguish low to high FI values over the landscape and the mean (μ) and standard deviation (σ) of FI are shown next to each plot.
(TIF)

S6 Fig GIS plots of the Fisher information results computed from the 2012–2018 phytoplankton data. A blue to red color scheme and marker size gradient are used to distinguish low to high FI values over the landscape and the mean (μ) and standard deviation (σ) of FI are shown next to each plot.
(TIF)

S1 Data Excel file of the physicochemical data from 1996–2018. Separate tabs for each year provide the data collected at the sampling stations.
(XLSX)

S2 Data Phytoplankton data from 1996–2018. Separate tabs for each year provide the data collected at the sampling stations.
(XLS)

S1 File (DOCX)

## References

[1] Garmestani A, Twidwell D, Angeler DG, Sundstrom S, Barichievy C, Chaffin BC, et al. Panarchy: opportunities and challenges for ecosystem management. Front Ecol Environ. 2020;18(10):576–83. doi: 10.1002/fee.2264 33408590 PMC7784709

[2] Ahmad N, Derrible S, Eason T, Cabezas H. Using Fisher information to track stability in multivariate systems. R Soc Open Sci. 2016;3(11):160582. doi: 10.1098/rsos.160582 28018650 PMC5180148

[3] Eason T, Chuang W-C, Sundstrom S, Cabezas H. An Information Theory-Based Approach to Assessing Spatial Patterns in Complex Systems. Entropy. 2019;21(2):182. doi: 10.3390/e21020182 31402835 PMC6688651

[4] Holling CS. Resilience and stability of ecological systems. Annual review of ecology and systematics. 1973;4(1):1–23.

[5] Allen CR, Angeler DG, Chaffin BC, Twidwell D, Garmestani A. Resilience reconciled. Nature sustainability. 2019;2(10):898–900. doi: 10.1038/s41893-019-0401-4 33623828 PMC7898119

[6] Angeler DG, Allen CR. Quantifying resilience. J Appl Ecol. 2016;53(3):617–24.

[7] Holling CS, Gunderson LH. Panarchy: understanding transformations in human and natural systems: Washington, DC: Island Press; 2002.

[8] Garmestani AS, Allen CR, Gunderson L. Panarchy: discontinuities reveal similarities in the dynamic system structure of ecological and social systems. Ecol Soc. 2009;14(1).

[9] Eason T, Cabezas H. Evaluating the sustainability of a regional system using Fisher information in the San Luis Basin, Colorado. J Environ Manage. 2012;94(1):41–9. doi: 10.1016/j.jenvman.2011.08.003 21930337

[10] Eason T, Garmestani AS. Cross-scale dynamics of a regional urban system through time. Region et Developpement. 2012;36:55–77.

[11] Eason T, Garmestani AS, Cabezas H. Managing for resilience: early detection of regime shifts in complex systems. Clean Technol Envir. 2014;16(4):773–83.

[12] Eason T, Garmestani AS, Stow CA, Rojo C, Alvarez-Cobelas M, Cabezas H. Managing for resilience: an information theory-based approach to assessing ecosystems. J Appl Ecol. 2016;53(3):656–65.

[13] Eason T, S. Garmestani A. Cross-scale dynamics of a regional urban system through time 2012.

[14] Spanbauer TL, Allen CR, Angeler DG, Eason T, Fritz SC, Garmestani AS, et al. Prolonged instability prior to a regime shift. PLoS One. 2014;9(10):e108936. doi: 10.1371/journal.pone.0108936 25280010 PMC4184814

[15] Spanbauer TL, Allen CR, Angeler DG, Eason T, Fritz SC, Garmestani AS, et al. Body size distributions signal a regime shift in a lake ecosystem. Proc Biol Sci. 2016;283(1833). 10.1098/rspb.2016.0249 PMC4936026 27335415

[16] Sundstrom SM, Angeler DG, Barichievy C, Eason T, Garmestani A, Gunderson L, et al. The distribution and role of functional abundance in cross-scale resilience. Ecology. 2018;99(11):2421–32. doi: 10.1002/ecy.2508 30175443 PMC6792002

[17] Sundstrom SM, Eason T, Nelson RJ, Angeler DG, Barichievy C, Garmestani AS, et al. Detecting spatial regimes in ecosystems. Ecol Lett. 2017;20(1):19–32. doi: 10.1111/ele.12709 28000431 PMC6141036

[18] Allen CR, Angeler DG, Cumming GS, Folke C, Twidwell D, Uden DR. Quantifying spatial resilience. J Appl Ecol. 2016;53(3):625–35.

[19] Mayfield RJ, Langdon PG, Doncaster CP, Dearing JA, Wang R, Nazarova LB, et al. Metrics of structural change as indicators of chironomid community stability in high latitude lakes. Quaternary Science Reviews. 2020;249:106594.

[20] Clements CF, Ozgul A. Indicators of transitions in biological systems. Ecology Letters. 2018;21(6):905–19. doi: 10.1111/ele.12948 29601665

[21] Dakos V, van Nes EH, Donangelo R, Fort H, Scheffer M. Spatial correlation as leading indicator of catastrophic shifts. Theor Ecol-Neth. 2010;3(3):163–74.

[22] Donangelo R, Fort H, Dakos V, Scheffer M, Van Nes EH. Early Warnings for Catastrophic Shifts in Ecosystems: Comparison between Spatial and Temporal Indicators. Int J Bifurcat Chaos. 2010;20(2):315–21.

[23] Eby S, Agrawal A, Majumder S, Dobson AP, Guttal V. Alternative stable states and spatial indicators of critical slowing down along a spatial gradient in a savanna ecosystem. Global Ecol Biogeogr. 2017;26(6):638–49.

[24] Guttal V, Jayaprakash C. Changing skewness: an early warning signal of regime shifts in ecosystems. Ecology Letters. 2008;11(5):450–60. doi: 10.1111/j.1461-0248.2008.01160.x 18279354

[25] Ratajczak Z, D’Odorico P, Nippert JB, Collins SL, Brunsell NA, Ravi S. Changes in spatial variance during a grassland to shrubland state transition. J Ecol. 2017;105(3):750–60.

[26] Roberts CP, Donovan VM, Allen CR, Angeler DG, Helzer C, Wedin D, et al. Monitoring for spatial regimes in rangelands. Rangeland Ecology & Management. 2021;74:114–8.

[27] Génin A, Majumder S, Sankaran S, Schneider FD, Danet A, Berdugo M, et al. Spatially heterogeneous stressors can alter the performance of indicators of regime shifts. Ecological Indicators. 2018.

[28] Kefi S, Guttal V, Brock WA, Carpenter SR, Ellison AM, Livina VN, et al. Early Warning Signals of Ecological Transitions: Methods for Spatial Patterns. Plos One. 2014;9(3):213–7. doi: 10.1371/journal.pone.0092097 24658137 PMC3962379

[29] Schneider FD, Kefi S. Spatially heterogeneous pressure raises risk of catastrophic shifts. Theor Ecol-Neth. 2016;9(2):207–17.

[30] Bury TM, Sujith R, Pavithran I, Scheffer M, Lenton TM, Anand M, et al. Deep learning for early warning signals of tipping points. Proceedings of the National Academy of Sciences. 2021;118(39). doi: 10.1073/pnas.2106140118 34544867 PMC8488604

[31] Buelo C, Carpenter S, Pace M. A modeling analysis of spatial statistical indicators of thresholds for algal blooms. Limnology and Oceanography Letters. 2018;3(5):384–92.

[32] Weissmann H, Kent R, Michael Y, Shnerb NM. Empirical analysis of vegetation dynamics and the possibility of a catastrophic desertification transition. Plos One. 2017;12(12). doi: 10.1371/journal.pone.0189058 29261678 PMC5737887

[33] Weissmann H, Shnerb NM. Predicting catastrophic shifts. J Theor Biol. 2016;397:128–34. doi: 10.1016/j.jtbi.2016.02.033 26970446

[34] Dai L, Vorselen D, Korolev KS, Gore J. Generic Indicators for Loss of Resilience Before a Tipping Point Leading to Population Collapse. Science. 2012;336(6085):1175–7. doi: 10.1126/science.1219805 22654061

[35] Rindi L, Dal Bello M, Dai L, Gore J, Benedetti-Cecchi L. Direct observation of increasing recovery length before collapse of a marine benthic ecosystem. Nat Ecol Evol. 2017;1(6). doi: 10.1038/s41559-017-0153 28812627

[36] Yin Z, Dekker SC, Rietkerk M, van den Hurk BJJM, Dijkstra HA. Network based early warning indicators of vegetation changes in a land-atmosphere model. Ecol Complex. 2016;26:68–78.

[37] Lindegren M, Dakos V, Groger JP, Gardmark A, Kornilovs G, Otto SA, et al. Early Detection of Ecosystem Regime Shifts: A Multiple Method Evaluation for Management Application. Plos One. 2012;7(7). doi: 10.1371/journal.pone.0038410 22808007 PMC3393716

[38] Scheffer M, Bascompte J, Brock WA, Brovkin V, Carpenter SR, Dakos V, et al. Early-warning signals for critical transitions. Nature. 2009;461(7260):53–9. doi: 10.1038/nature08227 19727193

[39] Roberts CP, Uden DR, Allen CR, Angeler DG, Powell LA, Allred BW, et al. Tracking spatial regimes in animal communities: Implications for resilience-based management. Ecological Indicators. 2022;136:108567.

[40] Angeler DG, Allen CR, Uden DR, Johnson RK. Spatial patterns and functional redundancies in a changing boreal lake landscape. Ecosystems. 2015;18(5):889–902.

[41] Fisher RA. On the mathematical foundations of theoretical statistics. Philosophical Transactions of the Royal Society of London Series A, Containing Papers of a Mathematical or Physical Character. 1922;222(594–604):309–68.

[42] Karunanithi AT, Cabezas H, Frieden BR, Pawlowski CW. Detection and Assessment of Ecosystem Regime Shifts from Fisher Information. Ecol Soc. 2008;13(1).

[43] González-Mejía A, Vance L, Eason T, Cabezas H. Recent developments in the application of Fisher information to sustainable environmental management. Assessing and Measuring Environmental Impact and Sustainability: Elsevier; 2015. p. 25–72.

[44] Vance L, Eason T, Cabezas H, Gorman ME. Toward a leading indicator of catastrophic shifts in complex systems: Assessing changing conditions in nation states. Heliyon. 2017;3(12):e00465. doi: 10.1016/j.heliyon.2017.e00465 29322097 PMC5753610

[45] Batty M. Spatial entropy. Geographical analysis. 1974;6(1):1–31.

[46] Gonzalez-Mejia AM, Eason TN, Cabezas H, Suidan MT. Social and economic sustainability of urban systems: comparative analysis of metropolitan statistical areas in Ohio, USA. Sustain Sci. 2014;9(2):217–28.

[47] Karunanithi AT, Garmestani AS, Eason T, Cabezas H. The characterization of socio-political instability, development and sustainability with Fisher information. Global Environ Chang. 2011;21(1):77–84.

[48] Mayer AL, Pawlowski CW, Cabezas H. Fisher information and dynamic regime changes in ecological systems. Ecol Model. 2006;195(1–2):72–82.

[49] Heino J, Alahuhta J, Bini LM, Cai Y, Heiskanen AS, Hellsten S, et al. Lakes in the era of global change: moving beyond single‐lake thinking in maintaining biodiversity and ecosystem services. Biological Reviews. 2021;96(1):89–106. doi: 10.1111/brv.12647 32869448

[50] Angeler DG, Allen CR, Barichievy C, Eason T, Garmestani AS, Graham NA, et al. Management applications of discontinuity theory. J Appl Ecol. 2016;53(3):688–98.

[51] Fried‐Petersen HB, Araya‐Ajoy YG, Futter MN, Angeler DG. Drivers of long‐term invertebrate community stability in changing Swedish lakes. Global change biology. 2020;26(3):1259–70. doi: 10.1111/gcb.14952 31808987 PMC7078863

[52] Olrik K, Blomqvist P, Brettum P, Cronberg G, Eloranta P. Methods for quantitative assessment of phytoplankton in freshwaters. Part I. 1998. Report No.: 4860.

[53] Blomqvist P, Herlitz E. Methods for quantitative assessment of phytoplankton in freshwaters. P. 2: Literature and its use for determination of planktic volvocales, tetrasporales, chlorococcales, and ulotrichales and formulas for calculation of biovolume of the organisms. Rapport-Naturvaardsverket (Sweden). 1998.

[54] Fölster J, Johnson RK, Futter MN, Wilander A. The Swedish monitoring of surface waters: 50 years of adaptive monitoring. Ambio. 2014;43(1):3–18. doi: 10.1007/s13280-014-0558-z 25403966 PMC4235935

[55] Fath BD, Cabezas H, Pawlowski CW. Regime changes in ecological systems: an information theory approach. J Theor Biol. 2003;222(4):517–30. doi: 10.1016/s0022-5193(03)00067-5 12781750

[56] Mayer AL, Pawlowski C, Fath BD, Cabezas H. Applications of Fisher information to the management of sustainable environmental systems. Exploratory data analysis using Fisher information: Springer; 2007. p. 217–44.

[57] Pawlowski CW, Cabezas H. Identification of regime shifts in time series using neighborhood statistics. Ecol Complex. 2008;5(1):30–6.

[58] Allen CR, Angeler DG, Garmestani AS, Gunderson LH, Holling CS. Panarchy: theory and application. Ecosystems. 2014;17(4):578–89.

[59] Gonzalez Mejia AM. Fisher information-Sustainability analysis of several US metropolitan statistical areas: University of Cincinnati; 2011.

[60] Gonzalez-Mejia AM, Eason TN, Cabezas H, Suidan MT. Assessing sustainability in real urban systems: the Greater Cincinnati Metropolitan Area in Ohio, Kentucky, and Indiana. Environ Sci Technol. 2012;46(17):9620–9. doi: 10.1021/es3007904 22775116

[61] Cabezas H, Eason T. Fisher information and order. Report. US EPA Development OoRa; 2010 3/15/2012. Report No.: EPA/600/R-10/182.

[62] Gonzalez-Mejia AM, Eason T, Cabezas H, Suidan MT. Computing and interpreting Fisher Information as a metric of sustainability: regime changes in the United States air quality. Clean Technol Envir. 2012;14(5):775–88.

[63] Veness C. Calculate distance, bearing and more between Latitude/Longitude points. 2017.

[64] Johnson RK, Hering D. Spatial congruency of benthic diatom, invertebrate, macrophyte, and fish assemblages in European streams. Ecol Appl. 2010;20(4):978–92. doi: 10.1890/08-1153.1 20597284

[65] Heino J. Are indicator groups and cross-taxon congruence useful for predicting biodiversity in aquatic ecosystems? Ecological Indicators. 2010;10(2):112–7.

[66] Lopes PM, Caliman A, Carneiro LS, Bini LM, Esteves FA, Farjalla V, et al. Concordance among assemblages of upland Amazonian lakes and the structuring role of spatial and environmental factors. Ecological Indicators. 2011;11(5):1171–6.

[67] Tolonen KT, Karjalainen J, Hämäläinen H, Nyholm K, Rahkola-Sorsa M, Cai Y, et al. Do the ecological drivers of lake littoral communities match and lead to congruence between organism groups? Aquatic Ecology. 2020;54(3):839–54.

[68] Angeler DG. Revealing a conservation challenge through partitioned long‐term beta diversity: increasing turnover and decreasing nestedness of boreal lake metacommunities. Diversity and Distributions. 2013;19(7):772–81.

[69] Prestes AC, Cacabelos E, Neto AI, Martins GM. Temporal stability in macroalgal assemblage standing stock despite high species turnover. Marine Ecology Progress Series. 2017;567:249–56.

[70] Huser BJ, Futter MN, Wang R, Fölster J. Persistent and widespread long-term phosphorus declines in Boreal lakes in Sweden. Science of the total environment. 2018;613:240–9. doi: 10.1016/j.scitotenv.2017.09.067 28915460

[71] Johnson RK, Goedkoop W, Sandin L. Spatial scale and ecological relationships between the macroinvertebrate communities of stony habitats of streams and lakes. Freshwater Biology. 2004;49(9):1179–94.

[72] Johnson RK, Angeler DG. Tracing recovery under changing climate: response of phytoplankton and invertebrate assemblages to decreased acidification. Journal of the North American Benthological Society. 2010;29(4):1472–90.

[73] Allen CR, Holling CS. Novelty, adaptive capacity, and resilience. Ecol Soc. 2010;15(3).

[74] Angeler DG, Allen CR, Johnson RK. Insight on invasions and resilience derived from spatiotemporal discontinuities of biomass at local and regional scales. Ecol Soc. 2012;17(2).

[75] Angeler DG, Allen CR, Johnson RK. Measuring the relative resilience of subarctic lakes to global change: redundancies of functions within and across temporal scales. J Appl Ecol. 2013;50(3):572–84.

[76] Roberts CP, Allen CR, Angeler DG, Twidwell D. Shifting avian spatial regimes in a changing climate. Nature Climate Change. 2019;9(7):562–6.

[77] Eklöf JS, Sundblad G, Erlandsson M, Donadi S, Hansen JP, Eriksson BK, et al. A spatial regime shift from predator to prey dominance in a large coastal ecosystem. Communications biology. 2020;3(1):1–9. doi: 10.1038/s42003-019-0734-6 32855431 PMC7452892

