# Supplementary figures and images for: Spatiotemporal variability in Swedish lake ecosystems
Source: PLoS One. 2022 Mar 21;17(3):e0265571. doi: 10.1371/journal.pone.0265571 (PMC8936495; doi:10.1371/journal.pone.0265571)

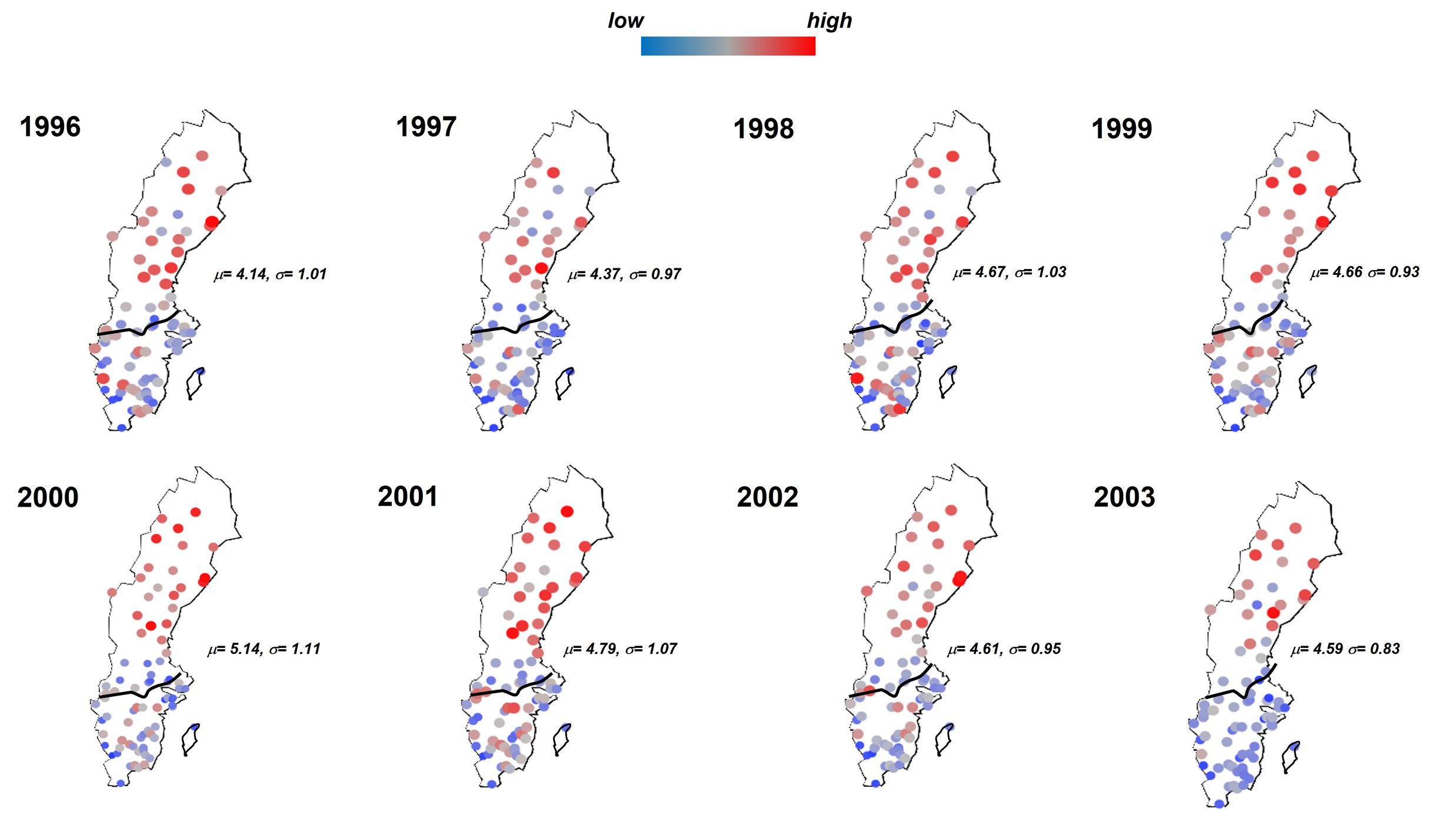

Supplement: S1 Fig — A blue to red color scheme and marker size gradient are used to distinguish low to high FI values over the landscape and the mean (μ) and standard deviation (σ) of FI are shown next to each plot. (TIF) [file pone.0265571.s001.tif]

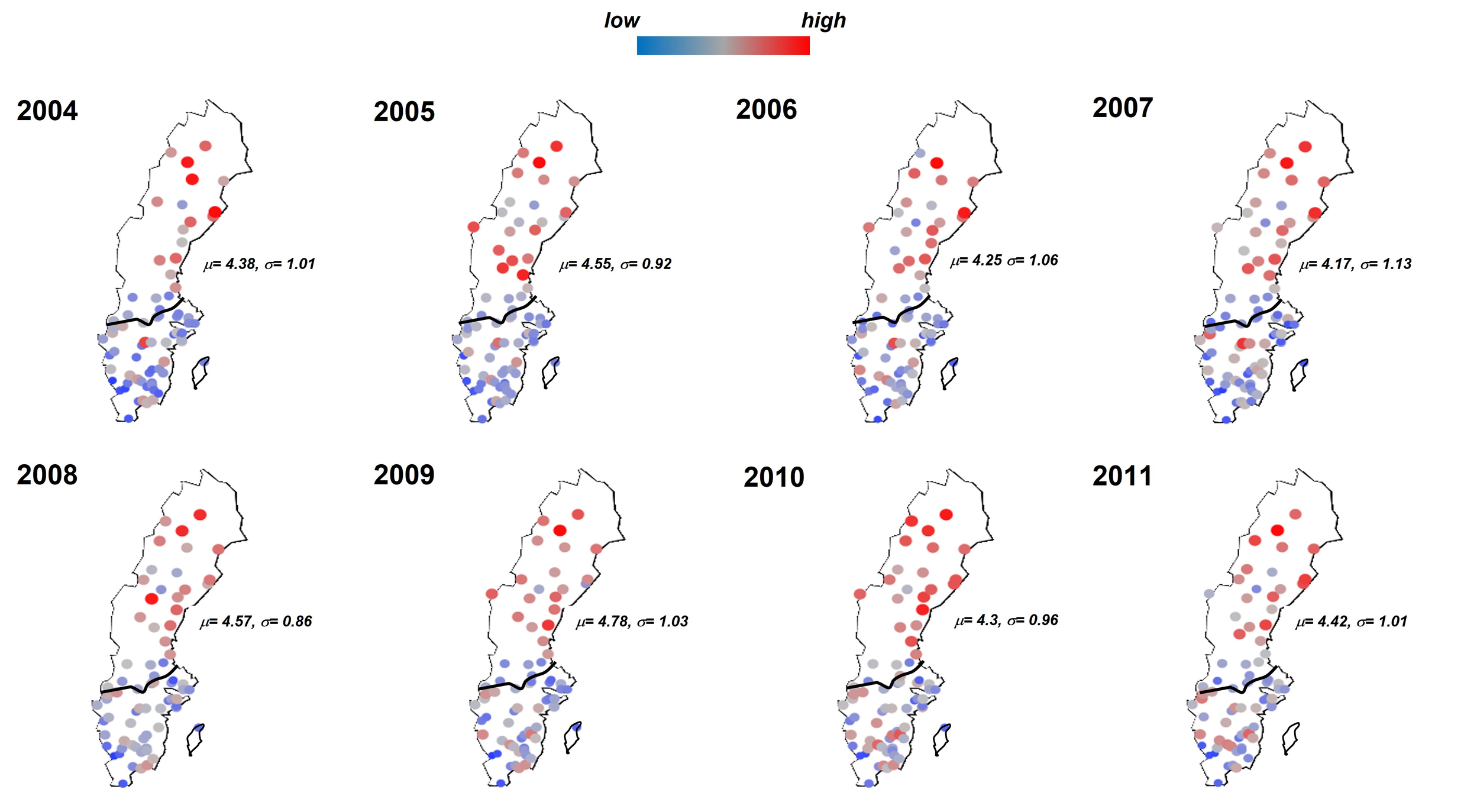

Supplement: S2 Fig — A blue to red color scheme and marker size gradient are used to distinguish low to high FI values over the landscape and the mean (μ) and standard deviation (σ) of FI are shown next to each plot. (TIF) [file pone.0265571.s002.tif]

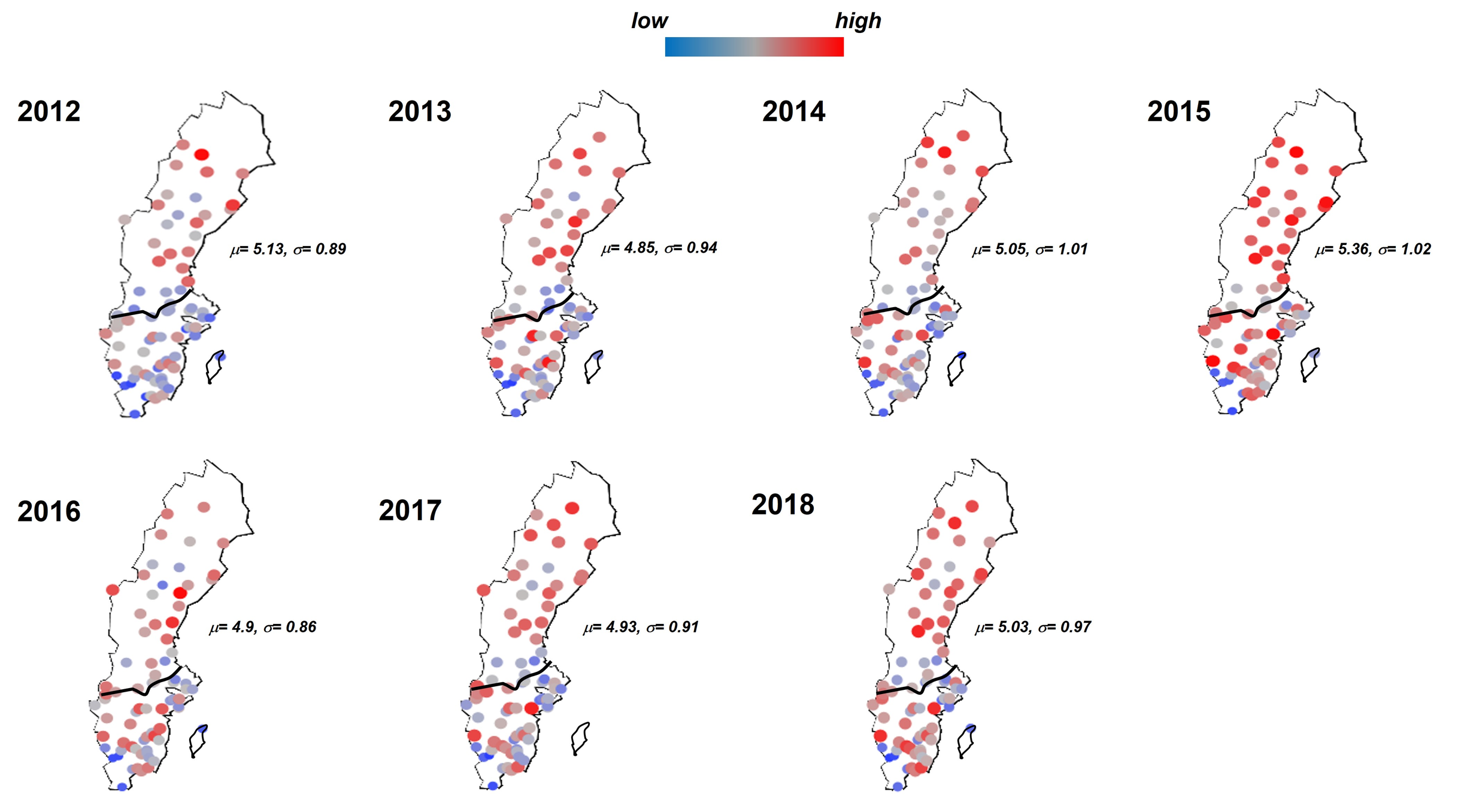

Supplement: S3 Fig — A blue to red color scheme and marker size gradient are used to distinguish low to high FI values over the landscape and the mean (μ) and standard deviation (σ) of FI are shown next to each plot. (TIF) [file pone.0265571.s003.tif]

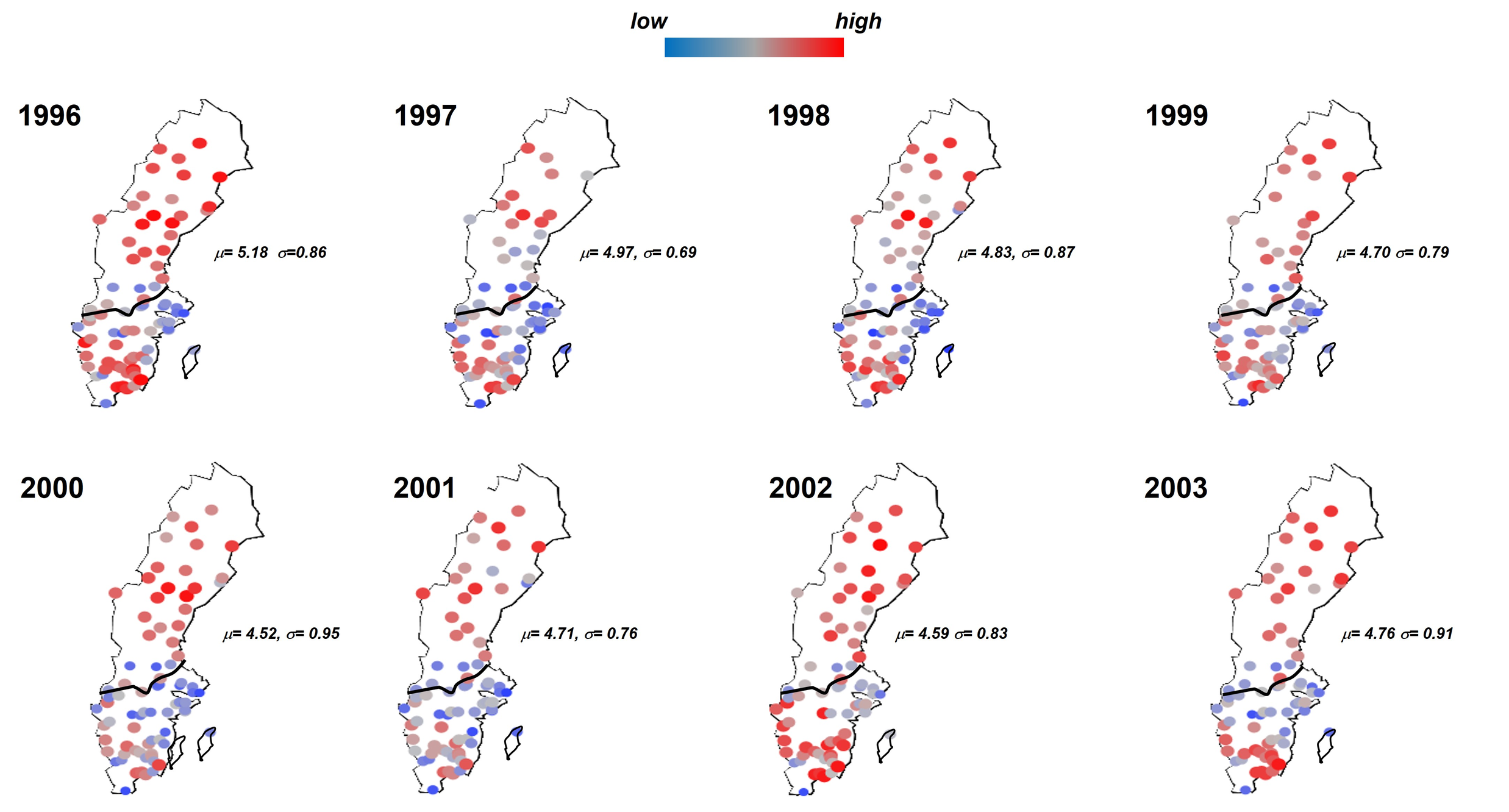

Supplement: S4 Fig — A blue to red color scheme and marker size gradient are used to distinguish low to high FI values over the landscape and the mean (μ) and standard deviation (σ) of FI are shown next to each plot. (TIF) [file pone.0265571.s004.tif]

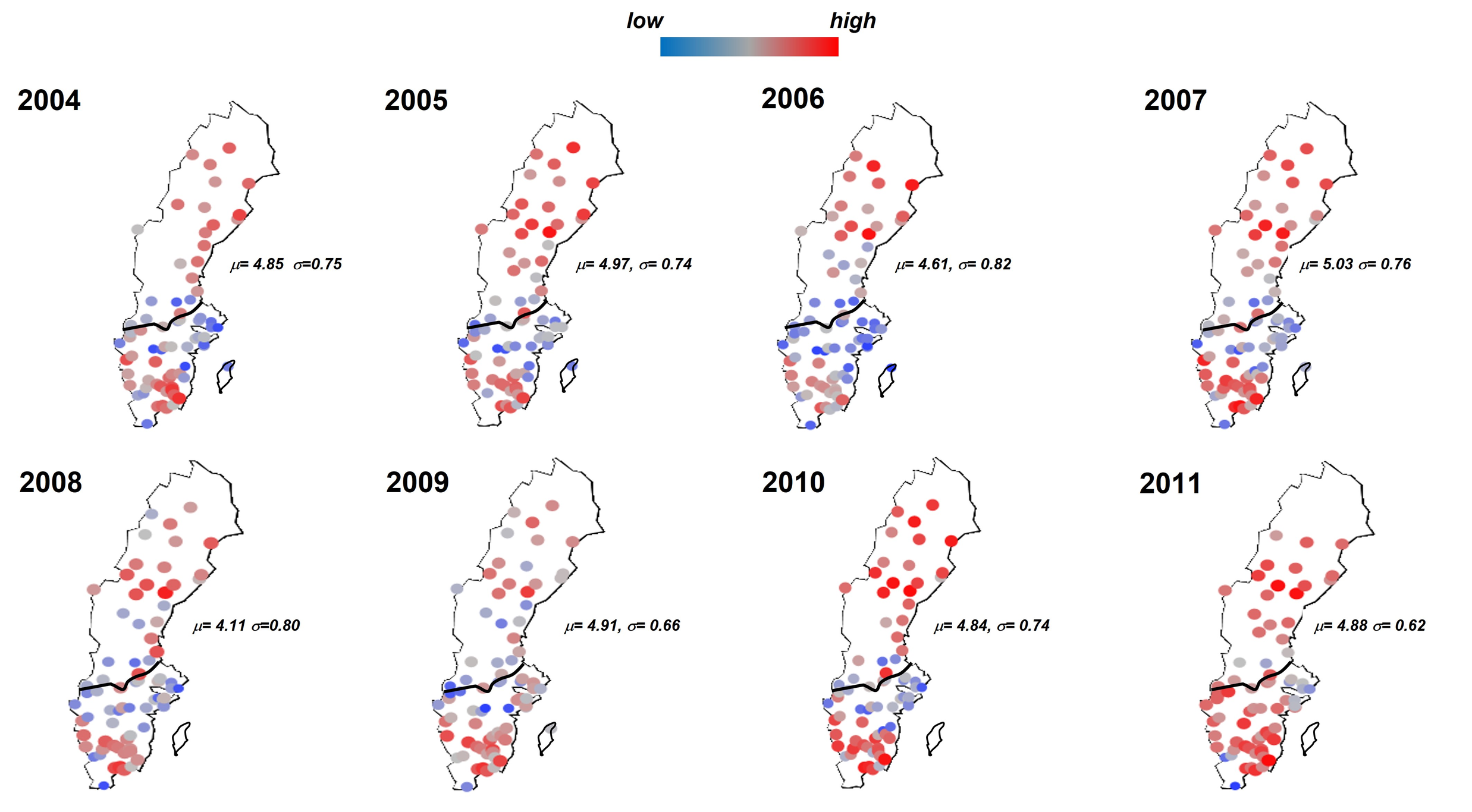

Supplement: S5 Fig — A blue to red color scheme and marker size gradient are used to distinguish low to high FI values over the landscape and the mean (μ) and standard deviation (σ) of FI are shown next to each plot. (TIF) [file pone.0265571.s005.tif]

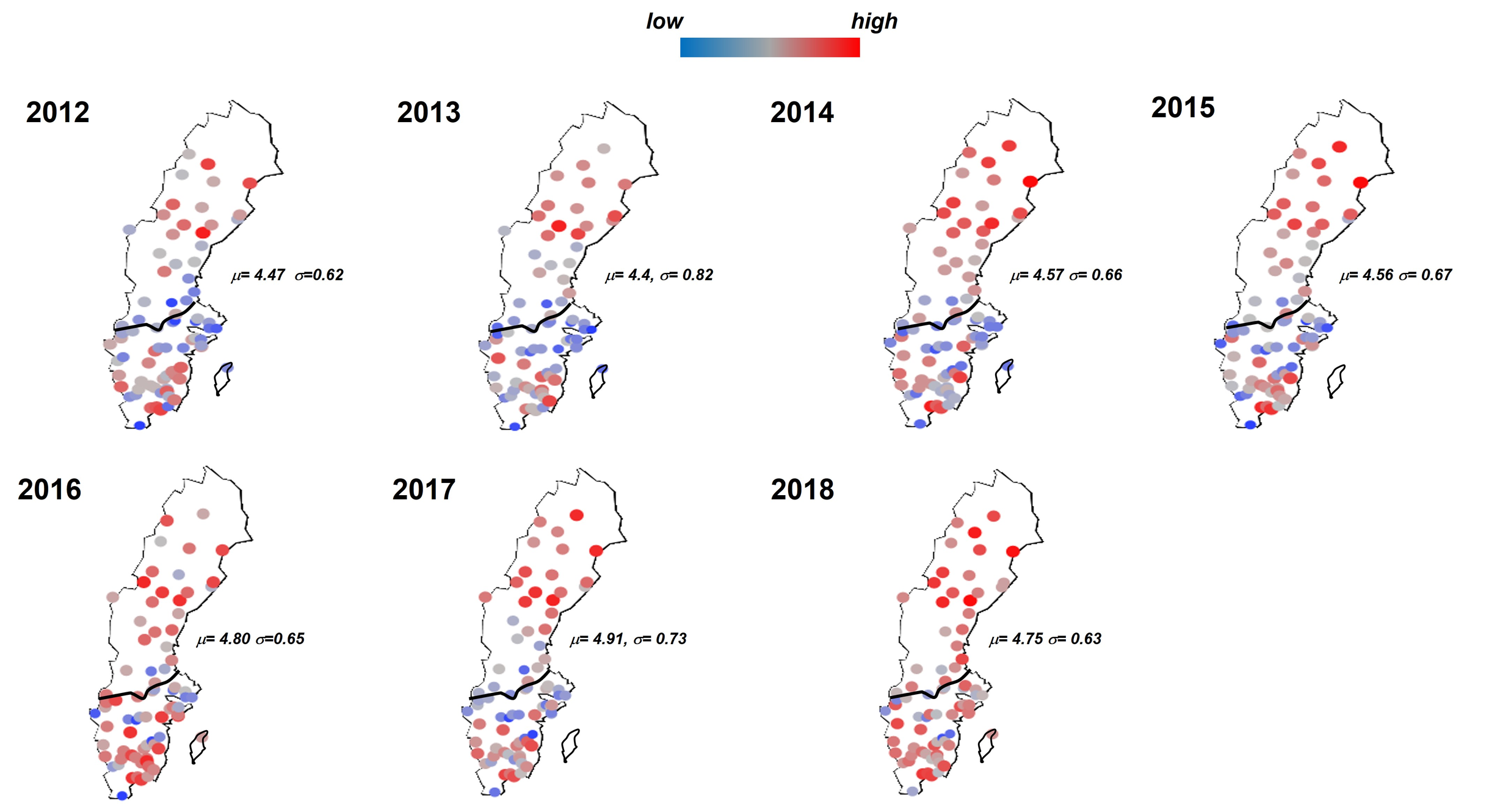

Supplement: S6 Fig — A blue to red color scheme and marker size gradient are used to distinguish low to high FI values over the landscape and the mean (μ) and standard deviation (σ) of FI are shown next to each plot. (TIF) [file pone.0265571.s006.tif]
