# Supplementary material for: Spatiotemporal variability in Swedish lake ecosystems
Source: PLoS One. 2022 Mar 21;17(3):e0265571. doi: 10.1371/journal.pone.0265571 (PMC8936495; doi:10.1371/journal.pone.0265571)
Supplement: S1 File — (DOCX) [file pone.0265571.s009.docx]

**Supporting Information**

GIS plots of Fisher information (FI) results computed from biophysical condition data from 1996-2018:

- Fig S1-S3: FI results from physicochemical condition (PC) data
- Fig S4-S6: FI results from phytoplankton (PHYTO) data


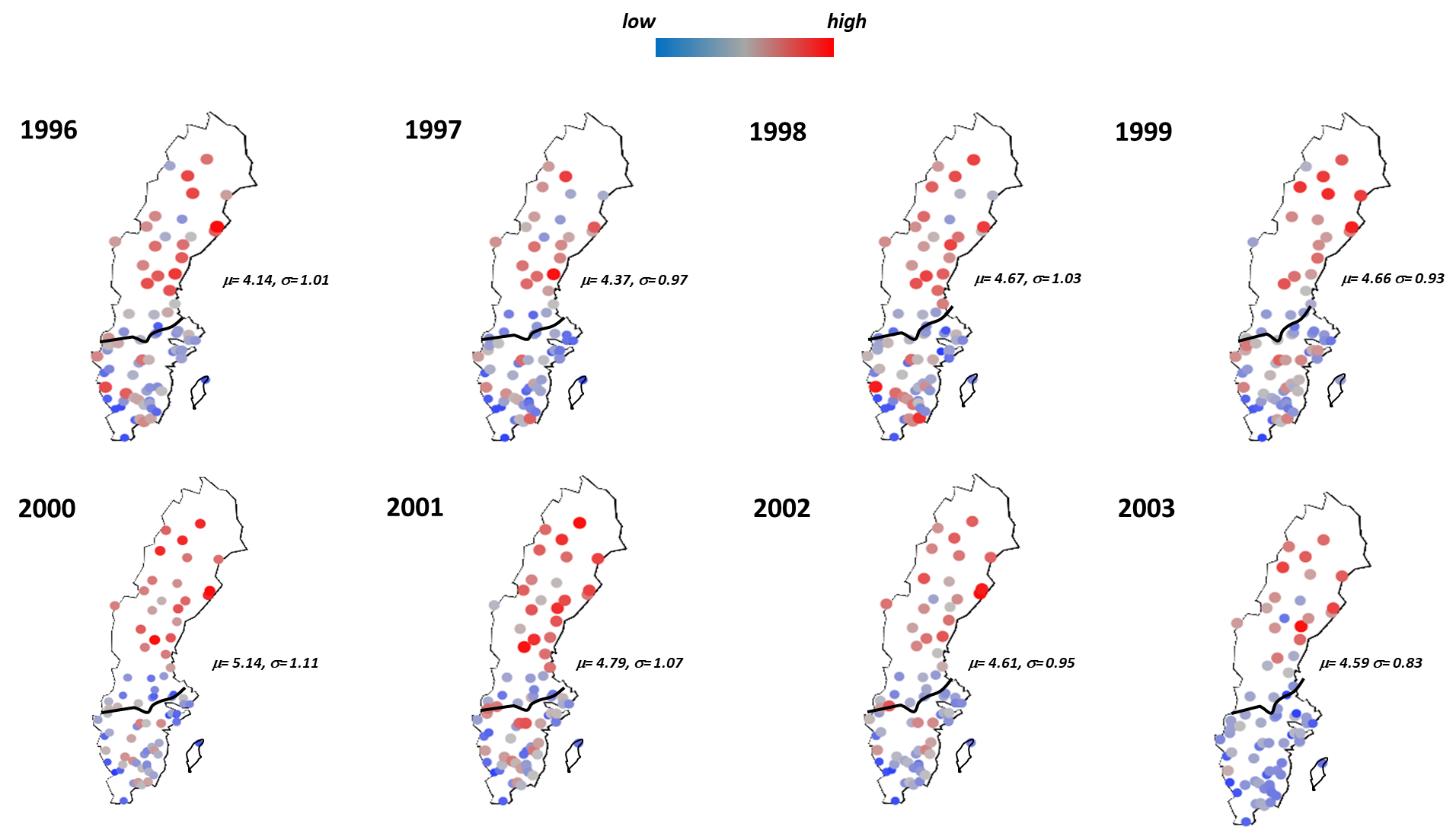


Fig S1. GIS plots of physicochemical condition FI (PC FI) computed from 1996-2003 data.


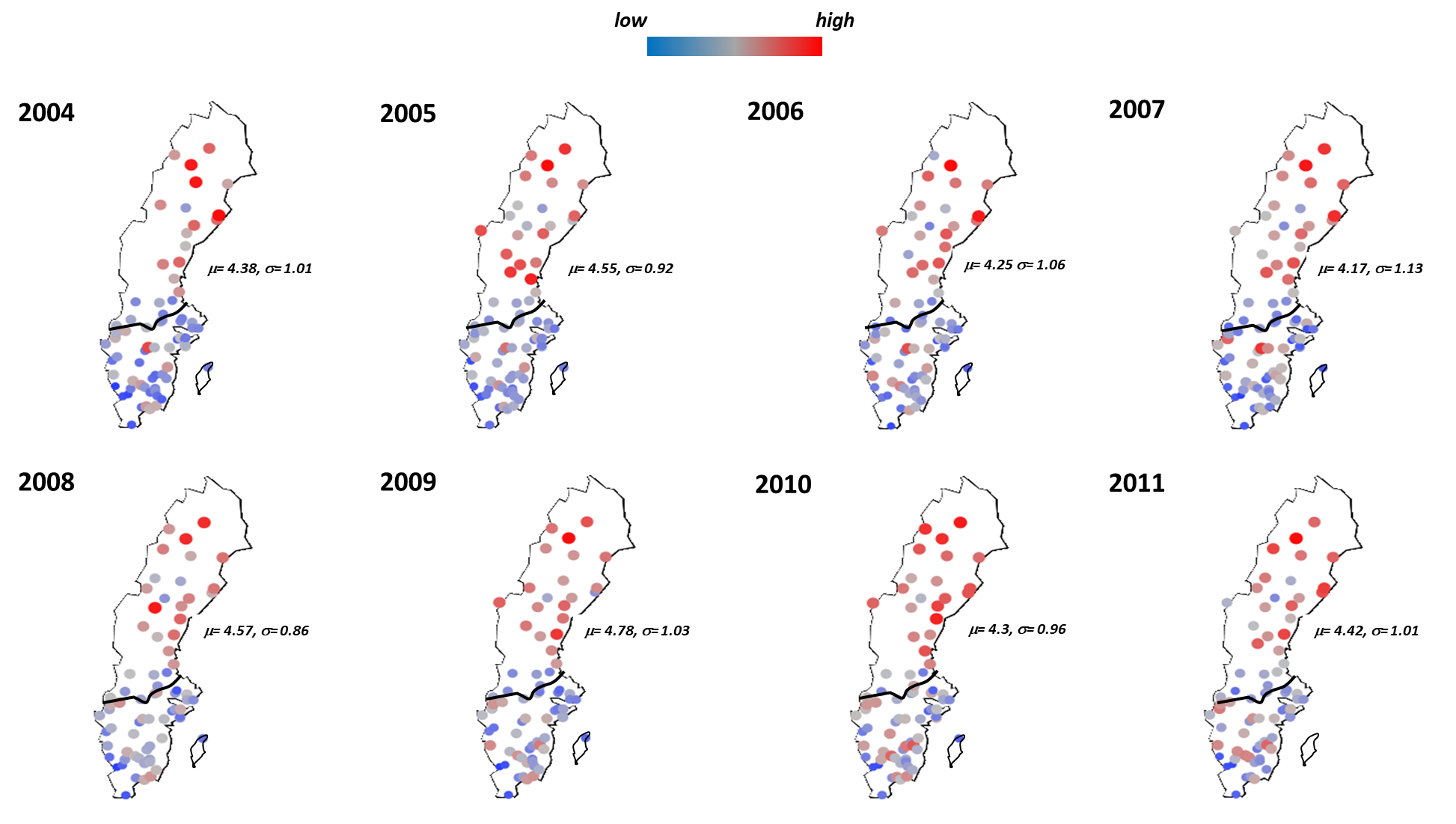


Fig S2. GIS plots of physicochemical condition FI (PC FI) computed from 2004-2011 data.


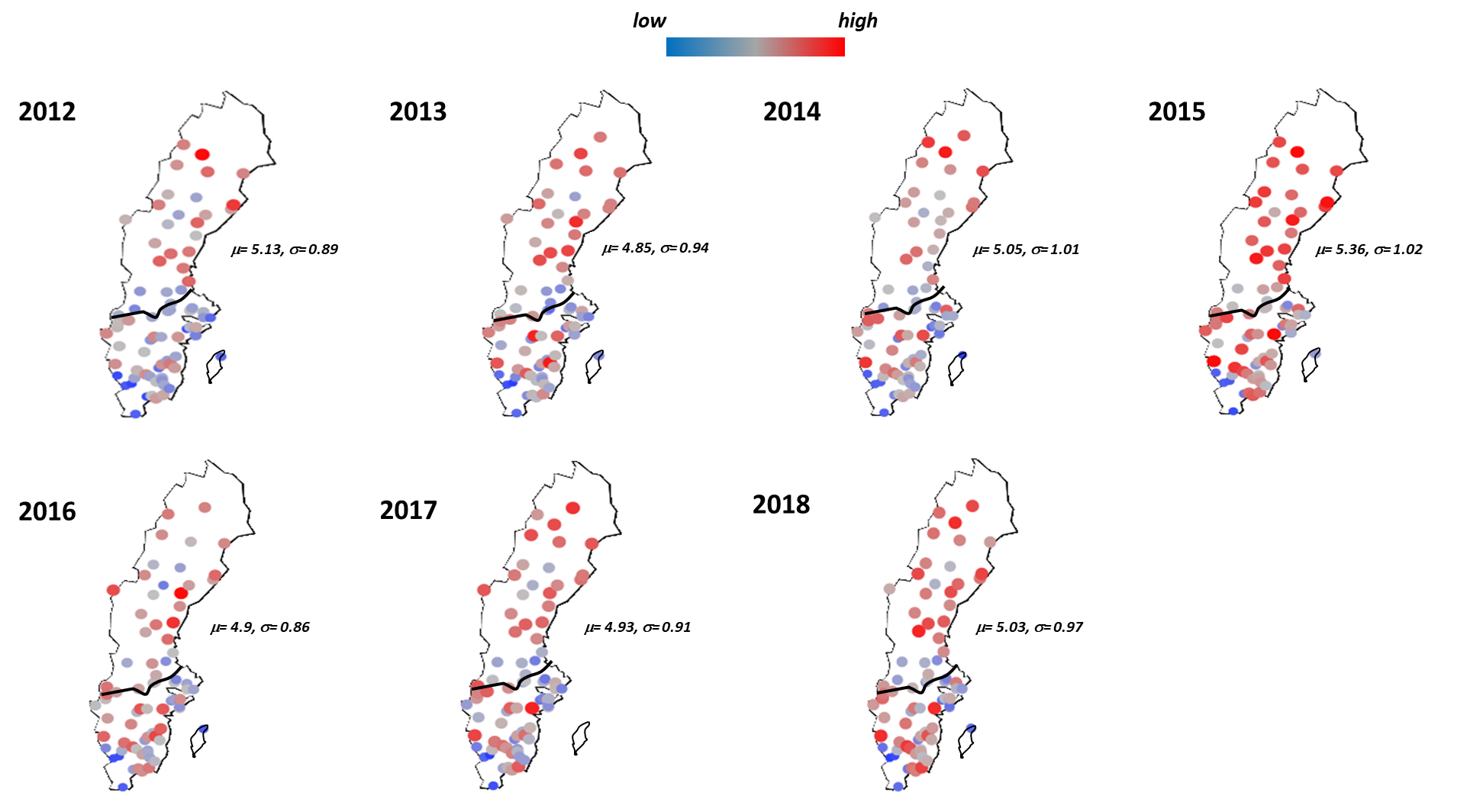


Fig S3. GIS plots of physicochemical condition FI (PC FI) computed from 2012-2018 data.


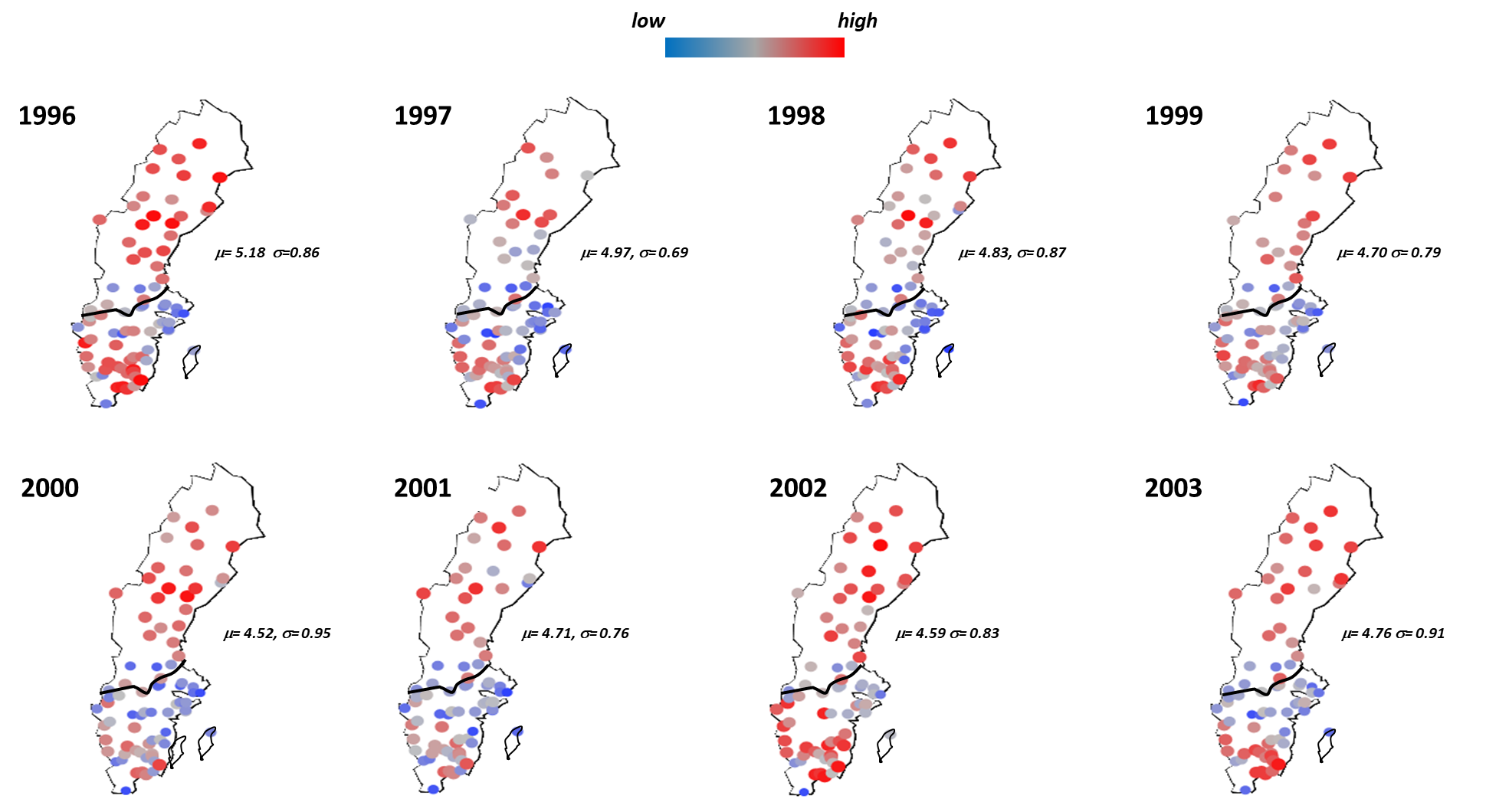


Fig S4. GIS plots of phytoplankton FI (PHYTO FI) computed from 1996-2003 data.


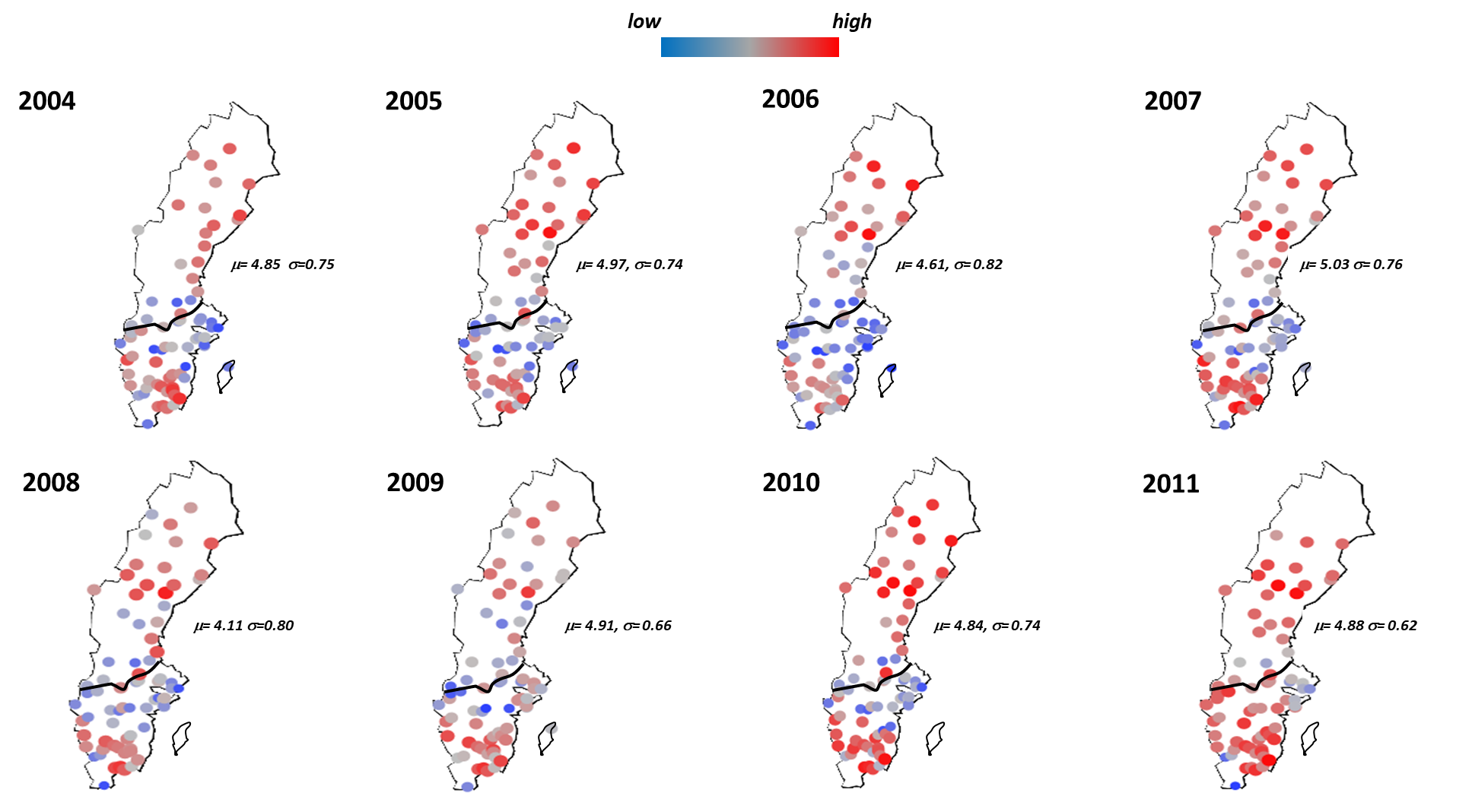


Fig S5. GIS plots of phytoplankton FI (PHYTO FI) computed from 2004-2011 data.


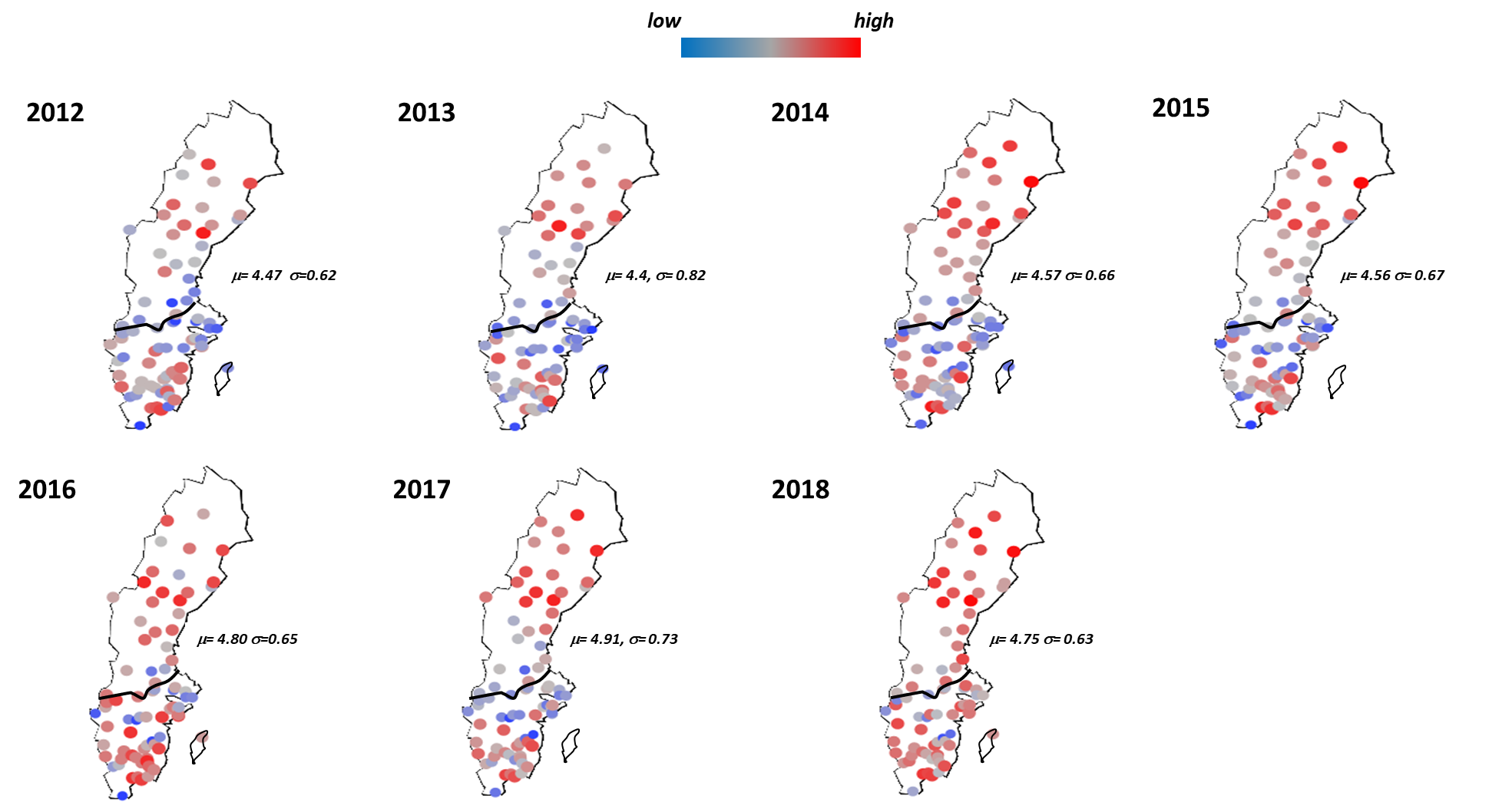


Fig S6. GIS plots of phytoplankton FI (PHYTO FI) computed from 2012-2018 data.
